# What values should an agent align with? An empirical comparison of general and context-specific values

**DOI:** 10.1007/s10458-022-09550-0

**Published:** 2022-03-28

**Authors:** Enrico Liscio, Michiel van der Meer, Luciano C. Siebert, Catholijn M. Jonker, Pradeep K. Murukannaiah

**Affiliations:** 1 Delft University of Technology: Technische Universiteit Delft, Delft, The Netherlands; 2 Leiden University: Universiteit Leiden, Leiden, The Netherlands

**Keywords:** Values, Ethics, Schwartz, Context, Axies, NLP

## Abstract

The pursuit of values drives human behavior and promotes cooperation. Existing research is focused on general values (e.g., Schwartz) that transcend contexts. However, context-specific values are necessary to (1) understand human decisions, and (2) engineer intelligent agents that can elicit and align with human values. We propose Axies, a hybrid (human and AI) methodology to identify context-specific values. Axies simplifies the abstract task of value identification as a guided value annotation process involving human annotators. Axies exploits the growing availability of value-laden text corpora and Natural Language Processing to assist the annotators in systematically identifying context-specific values. We evaluate Axies in a user study involving 80 human subjects. In our study, six annotators generate value lists for two timely and important contexts: Covid-19 measures and sustainable Energy. We employ two policy experts and 72 crowd workers to evaluate Axies value lists and compare them to a list of general (Schwartz) values. We find that Axies yields values that are (1) more context-specific than general values, (2) more suitable for value annotation than general values, and (3) independent of the people applying the methodology.

**Supplementary Information:**

The online version contains supplementary material available at 10.1007/s10458-022-09550-0.

## Introduction

Values are abstract ideals and our preferences among relevant and competing values guide our actions and attitude [1]. As agents operate in sociotechnical systems [2] on behalf of and among humans [3], agents’ behavior must accord with human values.

There is growing recognition [4–6] that values are central to robust and beneficial AI. In a value-sensitive AI system, an agent must first elicit or learn the value preferences of the stakeholders [7, 8]. Then, the agent can reason about aligning its actions with the values of the stakeholders [9–12]. However, a crucial question that must be answered before these steps is:

**What values** should an agent elicit, learn, or align with?

Several lists of *general values* have been proposed by ethicists [1, 13], political scientists [14], designers [15], and, recently, computer scientists [16]. These value lists aim to be applicable, broadly, across cultures and contexts. However, researchers recognize that not all values are relevant to all contexts [1, 17, 18]. Further, an individual’s preferences over general values may not be consistent across contexts [19]. That is, how we perceive and prioritize values is context dependent. For instance, one might value freedom over safety in general, but prioritize safety over freedom in the context of a global pandemic.

We define a *context-specific value* as a value that is applicable and defined specifically within a context. For example, in the context of information sharing on Social Media, privacy is an applicable value, but physical health is likely not (unless we are talking about the health effects of Computer Use, which is another context). Further, privacy can be interpreted as intruding one’s solitude, or control on information collection, processing, and dissemination [20]. Thus, privacy defined as one’s ability to control the extent to which her information is collected, processed, and disseminated is a value specific to the context of Social Media.

General values give insight into the broad behavioral tendencies of humans, such as openness to immigration and political activism [21]. However, for concrete applications, values must be situated within a context. Consider, for example, the task of value elicitation [17]—identifying individuals’ preferences over competing values—for the purpose of decision making on Green Energy Transition. Given this concrete task, we can elicit concerned users’ preferences between two context-specific values such as landscape preservation and energy independence or between two general values such as security and self-direction. We conjecture that the choice between the context-specific values is both easier for laypeople to express and more insightful for decision makers than the choice between the general values.

Other applications, where context-specific values can be beneficial, include: (1) communicating values to stakeholders [22], (2) translating values into design requirements [18, 23], (3) reasoning about conflicting values [9, 24], (4) synthesizing normative systems based on values [25–27], (5) investigating how values influence trust in agents [28, 29], and (6) verifying value adherence of an AI system [30].

How can we identify values specific to a context? Since values are (high-level) cognitive abstractions, human intelligence is necessary to conceptualize a value and reason about its relevance to a context. However, thinking about values is challenging even for humans [17, 18]. Thus, we need to systematically guide and assist humans in the process of identifying context-specific values.

We propose Axies (from the Greek word $\alpha \xi \acute{\iota } \epsilon \varsigma$, meaning *values*), a hybrid (human and AI) methodology to engage humans in identifying context-specific values and support the process via Natural Language Processing (NLP) techniques. A key idea behind Axies is to simplify the abstract task of value identification to a concrete task of value annotation given a (textual) value-laden opinion. With this approach, Axies enables human annotators to (1) learn about a context by exploring opinions about the context, and (2) think about values one opinion at a time.

**Fig. 1:**
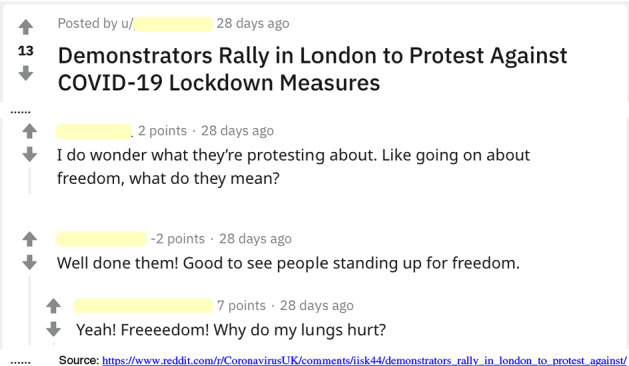
Example value-laden opinions on a Reddit forum

There is a growing availability of value-laden opinions for many contexts on the Web, e.g., on discussion forums, tweets, and blogs. For example, Fig. 1 shows examples of value-laden opinions on a Reddit discussion forum. By showing this opinion, Axies triggers a value annotator to think about the values of freedom and health in the context of Covid-19 measures. Value-laden opinions can also be collected by explicitly consulting a target population, e.g., [31].

Annotating a large opinion corpus is a significant effort. Axies distributes this task among a small group of annotators. Inspired by traditional coding methods such as the grounded theory method [32], the annotators engage in both divergent and convergent thinking by individually exploring the opinion corpus and collaboratively consolidating a value list. Axies employs an active learning strategy [33] to control the order in which opinions are shown to the annotators to reduce the annotation effort.

We conduct three experiments, involving 80 human subjects, to answer five research questions. Our experiments evaluate the characteristics of Axies values (i.e., values generated via Axies) and compare those with general (Schwartz) values [1].

- **Specificity** Are Axies values more *context-specific* than general values?
- **Comprehensibility** Are Axies values easier to *comprehend* than general values?
- **Consistency** Does Axies yield a *consistent* set of values, independent of the people applying the methodology?
- **Relationship** How do Axies values *relate* to general values?
- **Application** Are Axies values easier to *apply* than general values in the opinion annotation task?

In our first experiment, six annotators (in two groups of three) generate value lists specific to two contexts: Covid-19 relaxation measures, and sustainable Energy policies. In the second experiment, two policy experts evaluate the context-*specificity* of Axies and Schwartz value lists. Finally, in the third experiment, 72 crowd workers evaluate the *comprehensibility* of Axies and Schwartz value lists, and perform an annotation task with the value lists. From the crowd annotations, we (1) evaluate the *consistency* between Axies value lists generated by different annotator groups for the same context, (2) empirically study the *relationship* between Axies and Schwartz value lists, and (3) assess the *application* of the value lists by comparing the frequency and inter-rater reliability of value annotations.

**Contributions** (1) We propose Axies, a hybrid methodology to guide a group of human annotators in identifying context-specific values. Axies employs NLP techniques and active learning to engage the annotators in inducing values from an opinion corpus. (2) We conduct an experiment in which Axies is applied to generate four value lists in two contexts. (3) We perform two additional experiments to compare the Axies value lists and the Schwartz value list, quantitatively and qualitatively. These experiments provide valuable insights on what values (general vs. context-specific) to choose for engineering a concrete application and the associated trade-offs.

**Extension** This paper extends the conference paper from [34]. The two papers differ significantly in the evaluation. The conference paper does not include a comparative evaluation. In contrast, in this extension, we conduct additional experiments to compare Axies values with a baseline of general (Schwartz) values. In particular, we compare the context-specificity, comprehensibility and application of Schwartz and Axies value lists, finding significant differences as well as relationships between the two types of value lists. The comparative evaluation is a significant extension as it required new experiments (involving additional human subjects) and new quantitative and qualitative analyses, and it provides new insights. To the best of our knowledge, we conduct the first empirical study to systematically compare context-specific and general values. In addition, we expand the Related Works with recently published work, and reflect on the potential threats to the validity of our findings.

**Organization** Section 2 reviews related works. Section 3 describes Axies. Section 4 describes the experiments. Section 5 discusses our results. Section 6 concludes the paper. We include the study protocols and extended results in the appendix. We make the data publicly available [35]. The Axies web platform is separately described [36].

## Related works

We review works that attempt to estimate and identify values (Sects. 2.1 and 2.2). These works are closely related to our contribution. However, there is a large body of work on values in different computing subfields, including value-sensitive design, multiagent systems, and software engineering. We identify key works from these subfields to demonstrate the applications of our work (Sects. 2.3, 2.4, and 2.5).

### Value estimation

Values may not be explicitly referred to in day-to-day interactions. Often, they are expressed through language, behavior, and customs, and can vary significantly across people, socio-cultural environments, and contexts [11]. Thus, ascertaining values requires extensive personal communication and analysis. The burst of online communication and social media provides an unprecedented opportunity to study several social phenomena [37], including value understanding and estimation from language.

NLP techniques allow the (semi-)automatic estimation of values from text. Liu et al. [38] present a psychographic analysis of values based on users’ word use from e-commerce reviews. However, since moral values are often only implicit in language, automated extraction of values from text is challenging. Lin et al. [39] estimate moral values in tweets by combining textual features and background knowledge (context) from Wikipedia. Hoover et al. [40] use a Distributed Dictionary Representation [41] to study the expression of moral values in tweets about charitable donations posted during and after Hurricane Sandy. Several works [42–44] employ semi-automatic techniques to build value lexicons for facilitating the estimation of values in text.

The works above start from a general value list: Liu et al. [38] and Ponizovskiy et al. [44] use values from the Schwartz Value Survey [1]. Lin et al. [39], Hoover et al. [40], Araque et al. [42], and Hopp et al. [43] use the Moral Foundations Dictionary [14]. In contrast, our objective is to *identify* a value list specific to a context.

### Value identification

Boyd et al. [45] demonstrate that values learned from free-response language (e.g., Facebook status messages) yield better predictive coverage of real-world behavior than values extracted from self-report questionnaires such as Schwartz Value Survey. Building on [45], Wilson et al. [16] describe a crowd-powered algorithm to generate a hierarchy of general values. Teernstra et al. [46] demonstrate that a text classifier (of Twitter discussions) predicts values from Moral Foundations Theory more accurately than a hand-crafted dictionary of general value-related keywords.

Similar to the works above, we employ a data-driven approach towards values. Unlike these approaches (which consider general values), we focus on context-specific values essential for concrete use and analysis of values as argued by an increasing body of literature, e.g., [9, 17, 18, 22–27, 30, 47].

### Value Sensitive Design

Value identification is central to Value Sensitive Design (VSD) [15], a broad set of methods to design technology that accounts for human values. VSD includes methods for identifying value sources, representing values, and resolving value tensions. The VSD framework includes a general set of values relevant to all design tasks [15]. Then, stakeholders’ value preferences are elicited through techniques such as Value Scenarios [48], Value Dams and Flows [49], and Envisioning Cards [50].

Pommeranz et al. [18] recognize the instantiation of abstract values in specific contexts as an essential step in the effective realization of VSD. They acknowledge the need for self-reflection triggers since reflecting on values is not natural to most people. Axies fills the gaps in VSD Pommeranz et al. [18] recognize. First, Axies targets the identification of context-specific values. Second, Axies provides concrete triggers to humans (who need not be design experts) for reflecting on values.

### Values in engineering multiagent systems

Values are garnering increasing attention in engineering intelligent agents [5] and multiagent systems [2]. For instance, Mosca and Such [47] propose an agent that supports the value of privacy and identifies the optimal data sharing policy by considering the value preferences of users. Mehrotra et al. [29] investigate how human and agent value similarity influences a human’s trust in that agent. Chhogyal et al. [28] propose a method to assess trust between agents based on values. Serramia et al. [26, 51] employ value preferences to select the most value-aligned norm system. Montes and Sierra [25] automate the synthesis of normative systems based on value promotion. Tubella et al. [30] propose the *Glass-Box* approach to evaluate the moral bounds of an AI system by mapping values to norms that constrain inputs and outputs. Axies is intended to provide the input for such works, by identifying the values that are to be operationalized in the application context.

### Values in software engineering

Several researchers recognize that human values ought to be considered when engineering software [52–54]. Perera et al. [55] offer an overview of the prevalence of human values in recent Software Engineering (SE) publications. Values of stakeholders can often be elicited in the Requirement Engineering (RE) phase. Detweiler and Harbers [56] provide tools to elicit values and embed them in the RE process by collecting value-based user stories. Thew and Sutcliffe [57] elicit stakeholders’ values by linking them to their motivations and emotions. van de Poel [23] proposes a strategy for translating the elicited values into norms and design requirements.

Other works attempt to include values throughout the SE process. For example, Winter et al. [58] propose *Values Q-Sort*, a systematic approach for the elicitation and representation of values across the SE process. Perera et al. [59] introduce *Continual Value(s) Assessment*, a framework that elicits and tracks values throughout the SE process by modelling them as goals. However, such works typically employ existing value taxonomies (e.g., Schwartz’s [1] or Rescher’s [60]) to elicit stakeholders’ values. In our work, we aim to *identify* a value list relevant to a context. Then, the SE process for applications in a context can use the value list systematically identified for that context instead of general values.

## Axies methodology

**Fig. 2:**
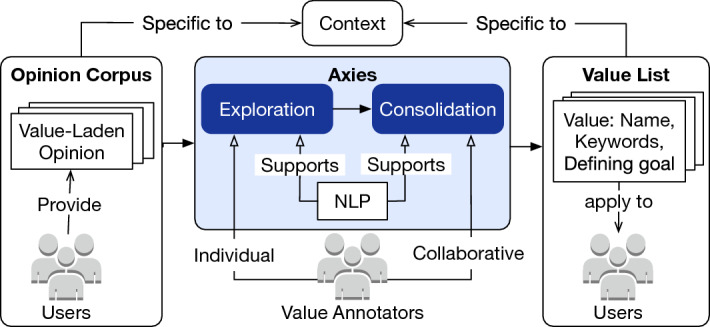
Overview of the Axies methodology

Figure 2 shows an overview of the Axies methodology. Given a context-specific opinion corpus, Axies yields a context-specific value list applicable to the *users* producing the opinion corpus. To do so, Axies (1) exploits NLP techniques and active learning, and (2) engages a group of value *annotators* in the systematic steps of exploration (individual) and consolidation (collaborative).

### Opinion corpus

The input to Axies is a corpus of users’ opinions within a context. Axies requires the corpus to include *value-laden* opinions. A value-laden opinion indicates a user’s value, explicitly or implicitly. For example, in Fig. 1 the value of freedom is explicitly mentioned but health is an implicit value.

#### Participatory Value Evaluation (PVE)

We construct the opinion corpora for Axies evaluation (Sect. 4) using data from PVE. A PVE elicits citizens’ preferences about government policy options [31]. Specifically, participants are offered a predetermined set of policy options, and informed about impacts. Then, participants are to advise their preferred portfolio of options while respecting the constraints of the government, and (optionally) provide motivations for their choices.

A PVE participant’s motivation is included as an opinion in our corpus. Often, these opinions offer valuable insights into the values of PVE participants. Table 1 shows examples of value-laden opinions of participants in a recent PVE on Covid-19 relaxation measures in the Netherlands [31].

**Table 1:** Examples of value-laden opinions in a Covid-19 PVE [31]

Preference	Motivation
Nursing homes allow visitors again	Loneliness and isolation are a bigger killer than Corona
All restrictions are lifted for persons who are immune	Someone’s got to keep the economy going

### Value list

The output of Axies is a *value list* specific to the context in which an opinion corpus is produced, and applicable to the users producing the corpus. We represent each value in the list by a name, a set of *keywords* that characterize the value in the context, and a *defining goal* [1] that specifies what “holding a value" means in that context. For instance, Table 2 shows examples of Covid-19 specific values, applicable to Dutch citizens, produced in the Axies evaluation.

**Table 2:** Examples of Dutch citizens’ Covid-19 values

Name	Keywords	Defining goal
Mental health	Loneliness, quality of life, stress	The strive towards protecting and improving one’s emotional and psychological well-being
Economic prosperity	Economy, stability, bankruptcy	Being able to pay and afford what you need

### Value annotators

Axies is intended to be executed by a small group of annotators, who (1) produce individual value lists during *exploration*, and (2) collaboratively merge the individual lists during *consolidation*.

Axies facilitates *inductive reasoning* in that the annotators infer values held by users (theory) based on the opinions users express (evidence). A key advantage of this inductive approach is that Axies yields values grounded in data. In addition, the inductive process provides an opportunity to systematically guide the annotators.

### Axies: value exploration

In the exploration phase, each annotator independently develops a value list (with name and keywords for each value) by analyzing users’ opinions. Depending on the context, opinion corpora can be quite large. For example, the Covid-19 opinion corpus [31] we evaluate contains about 60,000 opinions. Thus, it is not feasible for an annotator to analyze each opinion in a corpus.

Axies seeks to (1) reduce the number of opinions each annotator analyzes to produce a stable value list, and (2) increase the coverage of opinions (with respect to the corpus) the group of annotators analyze. To achieve these objectives, Axies employs NLP and active learning techniques to control the order in which the opinions in the corpus are exposed to the annotators. Thus, each annotator analyzes only a subset of the opinions in the corpus.

#### Opinion and value embeddings

Axies represents opinions and values as vectors computed from the Sentence-BERT [61] sentence embedding model *M*, which takes a word or a sentence as input and returns its vector representation in an *n*-dimensional space ($n=768$, in our case). In our experiments, we use the pre-trained bert-base-nli-mean-tokens model.

Let *M*(*o*) be the vector representation of an opinion *o*. Let $n_v$ be the name and $K_v = \{k_v^1, \dots , k_v^n$} be the set of keywords of a value *v*. Then, Axies computes the value vector *M*(*v*) using the Distributed Dictionary Representation [41] as:

1 $$\begin{aligned} M(v) = \frac{M(n_v) + {\sum _{k \in K_v} M(k)}}{|| M(n_v) + {\sum _{k \in K_v} M(k)}||}. \end{aligned}$$

With the vector representations, we can compute cosine similarity between values and opinions during opinion selection.

#### Exploration procedure

Let *A* be a set of value annotators for a context. Then, each annotator $a \in A$ follows the exploration steps below.

**Opinion selection** Axies employs an active learning technique known as *Farthest First Traversal* (FFT) [33, 62]. Using FFT, Axies selects opinions such that an opinion shown to an annotator *a* is the farthest from the opinions already shown to the annotators in group *A* and the values already annotated by the annotator *a*. Algorithm 1 shows the pseudocode for selecting an opinion to show an annotator *a*. We run one instance of this algorithm to select opinions for all annotators in *A* to reduce the overlap in opinions shown to different annotators in *A* (thereby, increasing the coverage of opinions shown to the annotators in *A*). However, for each annotator $a \in A$, we employ the individual value list, $V_a$.

**Figure:**
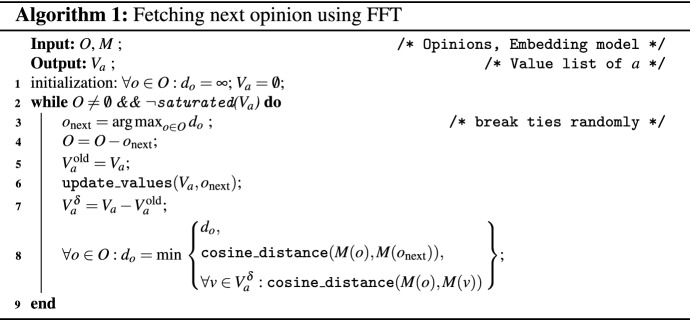

**Annotation** Algorithm 1 shows opinions to an annotator, sequentially. After seeing an opinion, an annotator can add a value (with a name and keywords) or update the name or keywords of an existing value in their value list. The annotators are asked to reason about the values underlying a user’s opinion. However, the value name or keywords need not explicitly appear in the opinion. When an annotator adds a value name, we show as keyword suggestions to the annotator the five most similar words to the value name based on a counter-fitted word embedding model [63], trained to push synonyms closer and antonyms farther.

**Fig. 3:**
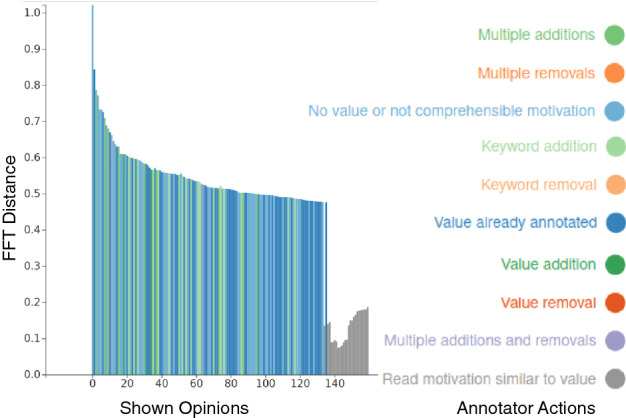
Example progress plot of exploration

**Termination** An annotator must judge when to stop annotating. We suggest the annotators to reach *inductive thematic saturation* [64], i.e., to continue annotation until the value list incurs no new changes for several new opinions shown to the annotator. We show a *progress plot* (similar to the example in Fig. 3) to assist the annotators in deciding on termination. The progress plot shows a bar for each opinion seen by an annotator; the length of the bar is the FFT distance ($d_o$) at which the opinion was fetched; and the bar color indicates the annotator’s action after seeing the opinion. A long sequence of opinions without addition of value names or keywords is an indicator of a stable value list.

**Refinement** Finally, Axies can fetch opinions similar to a value by computing cosine similarity between a value and the opinions not yet shown to an annotator. An annotator can fetch opinions similar to a value to refine the value, especially if it is not well formulated. Such a phase is visible in the final gray bars in Fig. 3.

### Axies: value consolidation

During consolidation, the annotators in a group collaborate to merge their individual value lists. Exploration and consolidation are complementary in that exploration facilitates divergent thinking whereas consolidation facilitates convergent thinking.

#### Consolidation procedure

To facilitate consolidation, Axies creates a combined value list, $V_A = \bigcup _{a \in A} V_a$ (the union of individual value lists of annotators in group *A*), and guides the annotators in systematically refining $V_A$.

**Value pairs** To simplify the consolidation process, Axies requires the annotators to consolidate only a pair of values at a time. Yet, consolidation is cognitively challenging. If performed naively, the annotators must compare all possible pairs of values in $V_A$, and repeat that process several times, to arrive at a refined $V_A$. To reduce the cognitive load, Axies controls the order in which value pairs are presented to the annotators—the most similar value pair from $V_A$ is shown first. This approach is beneficial because similar values are likely to be merged, reducing the size of $V_A$, which in turn, reduces the number of value pairs to consolidate.

**Consolidation actions** Given a pair of values, the original annotator of each value in the pair describes the value to the other annotators in the group. Axies can fetch the opinions that led to the annotation of a value to assist an annotator in recalling the reasoning behind the annotation. The annotators in the group discuss whether the two values are conceptually the same or distinct. Accordingly, the annotators can take one of the following actions.

- *Merge* the two values, if they are conceptually identical. The annotators may choose one of the two names or a new name for the merged value, and retain or update the keywords.
- *Update* one or both values, if the values are conceptually distinct, but changes in name or keywords make the distinction clearer.
- *Take no action*, if the two values are conceptually distinct, and the distinction is clear as is. If the annotators take no action for a pair of values, that pair is not shown to the annotators again even if that is the most similar value pair in $V_A$.

**Fig. 4:**
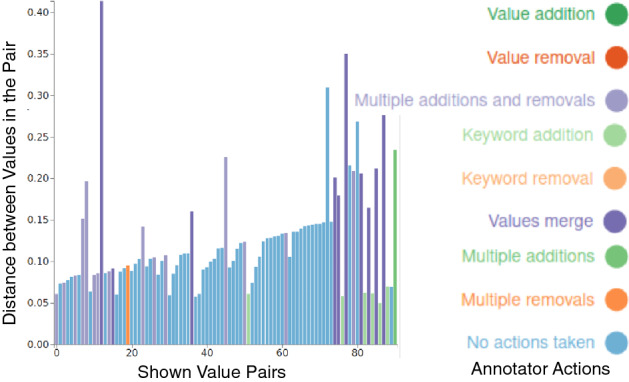
Example progress plot of consolidation

**Termination** Terminating consolidation is subject to annotators’ judgment as to whether the value list requires further refinement or not. Axies shows a plot (similar to Fig. 4) for the annotators to keep track of progress. As shown in the plot, the pairs of similar values shown early in the consolidation process lead to several value updates and merges. However, annotators may also manually choose values to merge or update; the intermittent spikes in Fig. 4 are due to such manual choices.

**Reflection** As the final step, the annotators critically reflect on the consolidated value list. In particular, Axies suggests the annotators to analyze each value in the list with respect to the main features of values. Schwartz [1] describes six main features of values; we include five of those, excluding the feature that (basic) values “transcend contexts” since Axies aims for context-specific values. During reflection, Axies also asks the annotators to add a defining goal for each value in the list. The defining goal characterizes what “holding a value" means. That is, a person holding a value in a context is likely to have the corresponding goal in that context. We defer the task of adding defining goals till the end of consolidation so that the task can be performed once for the final list of values.

## Experiments

**Fig. 5:**
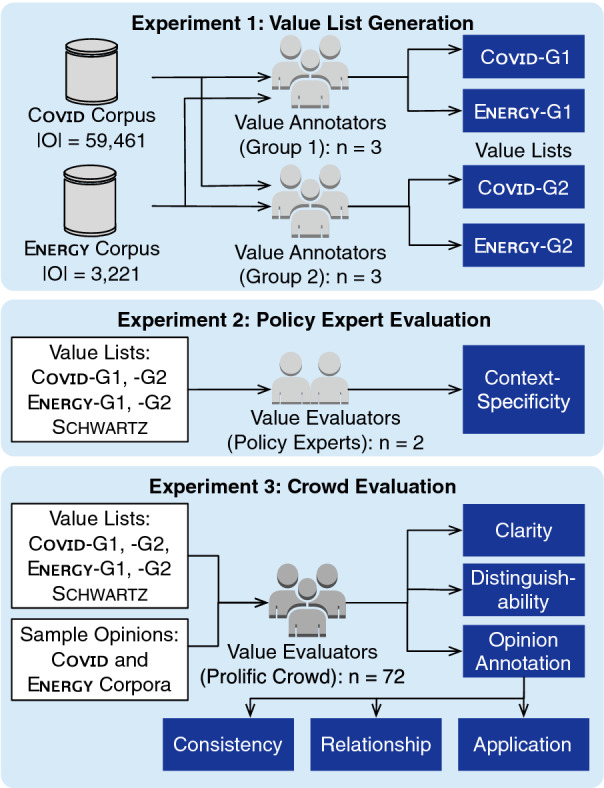
Overview of our experimental setup

We conducted three experiments, involving a total of 80 human subjects, to evaluate Axies as shown in Fig. 5. These experiments were approved by the Human Research Ethics Committee of the Delft University of Technology, and we received an informed consent from each subject.

In Experiment 1, two groups, G1 and G2, of three annotators each, employ Axies to generate value lists for two contexts (Covid and Energy) using a web application we developed [36]. Let the generated value lists be Covid-G1, Energy-G1, Covid-G2, and Energy-G2. We employ these lists and the full Schwartz list (ten values) [1] in the other two experiments to answer our research questions:

- **Specificity** In Experiment 2, we analyze the context-specificity of Covid (G1 and G2), Energy (G1 and G2), and Schwartz values.
- **Comprehensibility** In Experiment 3, we analyze the clarity of each value and the distinguishability between value pairs.
- **Consistency** In Experiment 3, we analyze the consistency between Covid-G1 and Covid-G2, and Energy-G1 and Energy-G2 using crowdsourced annotations.
- **Relationship** In Experiment 3, we use the annotations on a set of opinions to study the relationship between Axies and Schwartz values.
- **Application** In Experiment 3, we analyze the frequency of annotations and the annotator agreement to study the suitability of a value list for opinion annotation.

Through these experiments, we intend to evaluate the output of the Axies methodology. Thus, we compare the Axies (context-specific) values to the Schwartz list of (general) values due to its high contemporary influence [65]. We do not compare Axies to another value identification methodology since none of the existing methods (to the best of our knowledge) has the same purpose as Axies. Thus, the outputs of existing methods and Axies are not comparable. Most of the existing methods, e.g., [18, 48–50, 56–58], perform value elicitation, i.e., given an existing list of values, they identify an individual’s preferences over those values. In contrast, Axies performs context-specific value identification, i.e., given a context, Axies identifies the values relevant to that context. Among the related works, Wilson et al. [16] and Pommeranz et al. [18] are most similar to Axies. However, Wilson et al. [16] specifically pursue the creation of a general list of values. [18] work with context-specific values, but ultimately aim at eliciting individuals’ value preferences.

### Experiment 1: value lists

Four graduate students and two postdoctoral researchers, each working on a values-related research topic, participated as value annotators in Experiment 1. Two of these participants had a *technology and policy making* background, and four had a *computer science* background. The two groups, G1 and G2, were constructed to have one member with *technology and policy making* background and two members with a *computer science* background in each group.

#### Opinion corpora

We constructed two opinion corpora consisting of Dutch citizens’ opinions in two different contexts using data collected via PVE surveys.

**Covid** **corpus** contains opinions on *lifting* Covid*-19 measures in the Netherlands*. A PVE [31] for understanding participants’ preferences on lifting Covid-19 measures was conducted in the Netherlands during 29 April–6 May, 2020, when partial lockdown measures were in place in the Netherlands to limit the spread of Covid-19. The government had multiple plans for lifting such measures in the following weeks and months and wanted to gauge Dutch citizens’ opinions on the subject via PVE.

**Energy** **corpus** contains opinions on *future energy policies for the Súdwest Fryslân municipality* in the Netherlands. The municipality’s goal is to transition to renewable energy use, and there are multiple energy policies to achieve that goal. A PVE [66] was conducted during 10 April–3 May 2020, to understand Súdwest Fryslân residents’ opinions about the different energy policies.

The opinions in both Covid and Energy corpora were originally in Dutch. Since not all value annotators were fluent in Dutch, the opinions were translated to English using the MarianMT translator [67]. Further, opinions that contained only stop words or punctuation were removed. Then, the Covid corpus contained 59,461 and the Energy corpus contained 3,221 opinions.

### Experiment 2: context-specificity

Two graduate students with *technology and policy making* background participated in this experiment to evaluate the context-specificity of values. The two participants had performed the analogous experiment in the conference paper [34]. They were familiar with the Covid and Energy contexts in which the PVEs were conducted. However, these two participants were not involved in Experiment 1; thus, they did not know which value belonged to which list.

We created a value list $V_{CES}$ as the union of Covid-G1, Energy-G1, Covid-G2, Energy-G2, and Schwartz value lists. Then, for each value $v \in V_{CES}$, we asked each participant the extent to which they agree with the following statement (once for Covid and once for Energy context) on a Likert scale of 1 (strongly disagree) to 5 (strongly agree):

If I am a policy maker in the Covid (Energy) context, knowing citizens’ preferences about value *v* would help me in making a policy decision in that context.

We shuffled the combined value list $V_{CES}$ before asking the questions above so that each participant saw the values in a random order. For each value, we showed its name, keywords, and defining goal.

The two participants worked independently. After an initial round of ratings, the Intra-Class Correlation (ICC) between the two raters, an inter-rater reliability (IRR) metric for ordinal data [68], was 0.68. To ensure that the two participants had the same understanding of the task, they discussed their conceptual disagreements. Then, they performed another round of individual ratings, independently. The ICC after the second round was 0.74, which is considered just shy of excellent [68].

### Experiment 3: comprehensibility, consistency, relationship, and application

To evaluate the comprehensibility of values in a list, the consistency between Axies value lists for the same context, the relationship between Axies and Schwartz values, and the application of the value lists, we employed 72 Prolific^1^ crowd workers (including the 52 employed in the conference paper experiment [34]). The crowd workers were directed to the Axies web application to participate in the experiment.

Each crowd worker was assigned one value list and the corresponding context (in the case of the workers assigned the Schwartz list, half were assigned the Covid and half the Energy context). First, each worker was asked to read the information provided on the concept of values and on the corresponding context. Then, each worker performed three tasks.

#### Clarity

For each value in the list assigned to a worker, given the value name, keywords, and defining goal, the worker was asked the extent to which they agree with the following statement on a Likert scale of 1 (strongly disagree) to 5 (strongly agree):

The concept described by the value is clear.

#### Distinguishability

First, for a value list *V*, we computed the set $P_V$ of all value pairs: $\forall v_i, v_j \in V: v_i \ne v_j, \{ v_i, v_j\} \in P_V$. Then, we showed a subset of value pairs from $P_V$ (along with the respective keywords and defining goals) to each worker assigned to the list *V*. For each value pair shown, the worker was asked the extent to which they agree with the following statement on a Likert scale of 1 (strongly disagree) to 5 (strongly agree):

The two value concepts are distinguishable.

#### Opinion annotation

The final task for the crowd workers was to annotate opinions with values. First, we randomly selected 100 opinions from each opinion corpus. Then, we asked each worker assigned to a value list *V* to annotate a subset of the opinions selected for *V*’s context. For each opinion, a worker could select one or more values from *V* or mark the opinion as not value-laden.

We use the annotated opinions to measure the consistency of Axies value lists, the relationship between Axies and Schwartz values, and their application.

**Consistency** We use the opinion annotations for evaluating the consistency of Axies value lists. Since the same 100 opinions were annotated for both Axies value lists for a context, we can measure the association between values in the two lists based on the opinions annotated with those values. For example, if the same set of opinions are annotated with $v_1 \in$ Covid-G1 and $v_2 \in$ Covid-G2, then we consider $v_1$ and $v_2$ as closely associated. Then, we (qualitatively) assess the consistency between Covid-G1 and Covid-G2 (similarly, Energy-G1 and Energy-G2) based on the extent to which each value in one list (e.g., Covid-G1) is associated with one or more values in another list (e.g., Covid-G2).

**Relationship** We use the opinion annotations to study the relationship between Axies and Schwartz values. Analogous to the procedure described in the previous paragraph, we measure the association between Axies and Schwartz value lists based on the opinions annotated with those value lists.

**Application** We compute the frequency of annotations (the number of value annotations per opinion) and the inter-rater reliability (IRR) to measure the suitability of a value list for opinion annotation. We measure IRR via Fleiss’ Kappa [68] since the annotations were categorical and all opinions were rated by more than two workers.

#### Task distribution

Table 3 shows the number (#) of workers assigned to each value list, and the numbers of values, value pairs, and opinions assigned to each worker. The value list and the sets of value pairs and opinions were randomly assigned. The number of workers for each list was sufficient to obtain three annotations per opinion and three distinguishability ratings per value pair (one worker in each list annotated fewer than the shown number of pairs since that was sufficient to get three ratings per pair). Each worker rated the clarity of all values in the assigned list.

**Table 3:** Overview of the crowd task

Value list	#Workers	#Values	#Value pairs	#Opinions
Covid-G1	12	11	14	25
Covid-G2	10	9	11	30
Energy-G1	15	14	19	20
Energy-G2	15	13	16	20
Covid-Schwartz	10	10	7	30
Energy-Schwartz	10	10	7	30

#### Quality control

The crowd workers were required to be fluent in English and have submitted at least 100 tasks with at least 95% acceptance rate. We included four attention check questions: two in distinguishability rating and two in opinion annotation task.

A total of 115 workers completed the task. We included a worker’s task in our analysis only if the worker (1) passed both attention checks during distinguishability rating; and (2) at least one attention check during opinion annotation (we used one instead of two as the cut-off because there was some room for subjectivity in answering the two attention check questions asked during opinion annotation). These criteria were set before any analysis of crowd work was done. Of the 115 workers, 72 satisfied the criteria above.

We suggested the time required for task completion (liberal estimate) as 45 min. The mean time spent by a crowd worker on our task was 32 min (with 17 min standard deviation). Each worker was paid £5.6 (at the rate of £7.5 per hour).

### Statistical analyses

We perform the following statistical analyses on the data we collect.

1. To compare two ordinal samples, we employ Wilcoxon’s ranksum test (nonparametric) [69] at 5% significance level.
2. To compare two continuous samples, which meet the normality assumption, we employ Welch’s *t* test [70] at 5% significance level. If one of the samples does not meet the normality assumption, we employ the Wilcoxon’s ranksum test.
3. To compare more than two ordinal samples, we employ Kruskal-Wallis test (nonparametric extension of ANOVA) [69] at 5% significance level. When the Kruskal–Wallis test rejects the null hypothesis, we employ Dunn’s multiple comparison test [71] with the Holm–Bonferroni correction to compare pairs of samples.
4. To measure the effect sizes (the amount of difference) between pairs of ordinal or continuous samples, we employ Cliff’s Delta [72]. The Cliff’s Delta is positive when the values in the first sample are greater than the values in the second sample more often, and negative when the values in the first sample are less than the values in the second sample more often. The magnitude of the delta is estimated according to the suggested thresholds: $\delta <0.147$ is negligible (N); $\delta <0.33$ is small (S); $\delta <0.474$ is medium (M); and large (L), otherwise.

Other types of comparisons (e.g., comparisons of more than two continuous samples) are not applicable to the data we collect.

## Results and discussion

We discuss the main results from our three experiments in this section. Section 5.1 shows the value lists produced in Experiment 1. Sections 5.2, 5.3, 5.4, and 5.5 discuss results from Experiments 2 and 3, answering our five research questions.

### Value lists

#### Exploration

A total of 12 explorations (six per context) were performed. In the Covid context, the mean time for exploration was 69.17 min (SD 12.01 min), and the mean number of values annotated was 11.17 (SD 2.64). In the Energy context, the mean time for exploration was 67.5 min (SD 10.84 min), and the mean number of values annotated was 12.83 (SD 5.23).

#### Consolidation

A total of four consolidations were performed (two groups of three annotators each; two consolidations, one per context, for each group), producing four value lists. Table 4 presents an overview of the four value lists and the Schwartz value list [1] for comparison. The complete lists (including keywords and defining goals) are in the Appendix B.1.2. The times spent in consolidating Covid-G1, Energy-G1, Covid-G2, and Energy-G2 were 105, 110, 115, and 120 min, respectively.

**Table 4:** The value lists generated through Axies, and the Schwartz [1] value list

Context	List	Value names
Covid	G1	Well-being, Safety, Economic prosperity, Enjoyment, Fairness, Feasibility, Nuclear family, Autonomy, Care, Control
	G2	Mental health, Safety and health, Economic security, Acceptance of misbehavior, Pleasure, Conformity, Equality, Belonging to a group, Autonomy
Energy	G1	Community, Distributional justice, Innovation, Support, Guidance,Landscape preservation, Energy independence, Effectiveness,Sustainability, Planning for rainy days, Equal opportunities, Distrust, Regional benefits, Representation
	G2	Community, Initiative, Freedom, Organizational leadership,Involvement, Nature and landscape, Technical reliability,Technological innovation, Local benefit, Support,Free market economy, Inevitability, Fairness
General	Schwartz	Tradition, Conformity, Security, Power, Achievement, Hedonism,Stimulation, Self-Direction, Universalism, Benevolence

### Context-specificity

To evaluate the context-specificity of a value list, we measure the extent to which the values in a list can influence policy decisions in the context for which the list was produced compared to a value list produced for a different context and the Schwartz value list. We compute the specificity of a value *v* for a context *c*, as the mean of the ratings the two policy experts gave to value *v* for the context *c*. Recall that the policy experts were not aware of the context for which a value was annotated, a priori. The policy experts spent three hours each to rate the specificity of value lists.

**Fig. 6:**
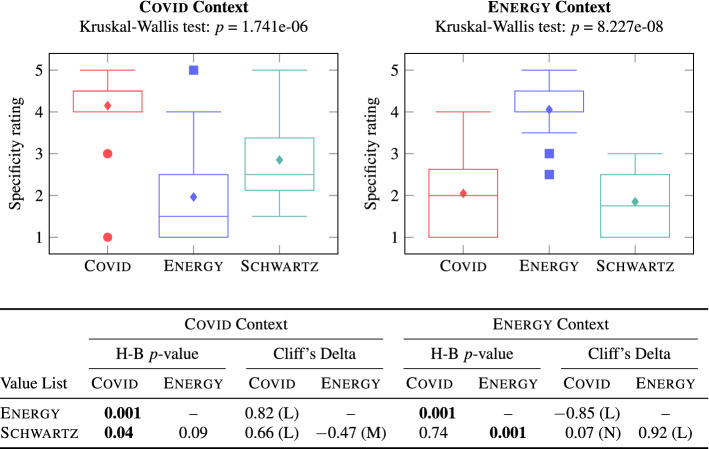
The context-specificity of Axies and Schwartz value lists

Figure 6 (left) compares the specificity of Covid (including G1 and G2), Energy (including G1 and G2), and Schwartz values for the Covid context. Figure 6 (right) compares the specificity of Covid (including G1 and G2), Energy (including G1 and G2), and Schwartz values for the Energy context.

Since the Kruskal–Wallis test indicated ($p<$ 0.05) that one of the three samples is significantly different from the others in both (left and right) comparisons in Fig. 6, we perform Dunn’s test to compare multiple pairs of samples. The table at the bottom of Fig. 6 shows the Holm–Bonferroni (H–B) corrected *p*-values as well as the effect sizes, measured via Cliff’s Delta, for each pairwise comparison. For each cell in the table, the first sample in the comparison is indicated in the column header and the second sample in the comparison is indicated in the row header.

First, we observe that, in the Covid context, Covid values have significantly higher specificity ratings than the Energy and Schwartz values with a large effect size. Similarly, in the Energy context, Energy values have significantly higher specificity ratings than the Covid and Schwartz values with a large effect size. This suggests that Axies values are more context-specific than Schwartz values. This is an important result since it demonstrates that the Axies methodology serves its purpose of producing context-specific value lists.

Second, the context-specificity varies among the values within the Axies lists. On the one hand, the specificity of a few Axies values is low. Specifically, Control (Covid), Representation, Technological Innovation, and Equal Opportunities (Energy) received average ratings lower than 3 for their respective context. We observe that these values are phrased broadly, and they may need refinement. On the other hand, the specificity of some Axies values was high for both contexts. Specifically, the Covid values of Control, Fairness, and Equality were rated higher than 3 for the Energy context. Similarly, the Energy values of Inevitability, Fairness, and Distrust were rated higher than 3 for the Covid context. Thus, some Axies values can be applicable to more than one context.

Finally, the specificity of Schwartz values can vary from one context to another. Specifically, the Schwartz values have higher specificity ratings in the Covid context than the Energy context. The nature of the two contexts can explain this difference—whereas the Covid context encompasses many aspects of life (at the moment of writing), the Energy context is narrower. Hence, in the latter case, the (general) Schwartz values are likely to be less informative.

### Comprehensibility

We employ crowdsourced data to evaluate the clarity of values and the distinguishability between value pairs in a list.

#### Clarity evaluation

**Fig. 7:**
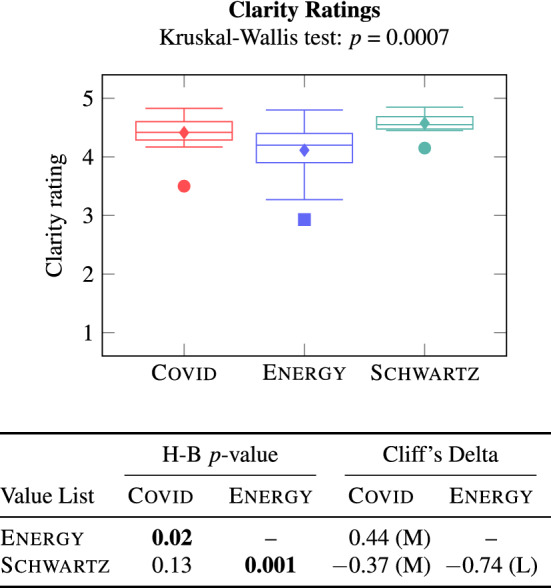
Clarity ratings of Axies and Schwartz values

Recall that the clarity of a value in a list was rated by each crowd worker assigned to that list, yielding at least ten clarity ratings (Table 3) per value. Figure 7 shows the distribution of mean clarity ratings of Covid, Energy, and Schwartz values.

First, the mean clarity rating of all but one Axies value (among values in all four lists) was at least 3. The Energy value of Distrust received the clarity rating of less than 3. The Distrust value has the defining goal “Big players (government, large companies) should not be in charge of solving problems on citizens’ behalf.” We conjecture that the connection between the Distrust value’s name and its defining goal is not obvious, and that is the reason for the value’s low clarity rating. However, a large majority (80.9%) of the Axies values received a mean clarity rating of at least 4. This suggests that Axies value lists are clear to end users.

Second, from the comparative evaluation, we observe no significant difference in the clarity of Covid and Schwartz values. However, the Covid and Schwartz values have significantly better clarity than the Energy values with a medium and a large effect size, respectively. A potential reason for the better clarity of Covid values compared to the Energy values is the timeliness of the Covid context. Since people are currently experiencing the pandemic, they can easily understand the values in this context. In contrast, the Energy context yields highly specialized values (e.g., Energy Independence) which may appear unclearer to a layperson. A potential reason for the better clarity of Schwartz values compared to Energy values (and Covid values although the difference is not statistically significant) is that the Schwartz values, being the result of years of refinement, are polished and easier to understand.

#### Distinguishability evaluation

**Fig. 8:**
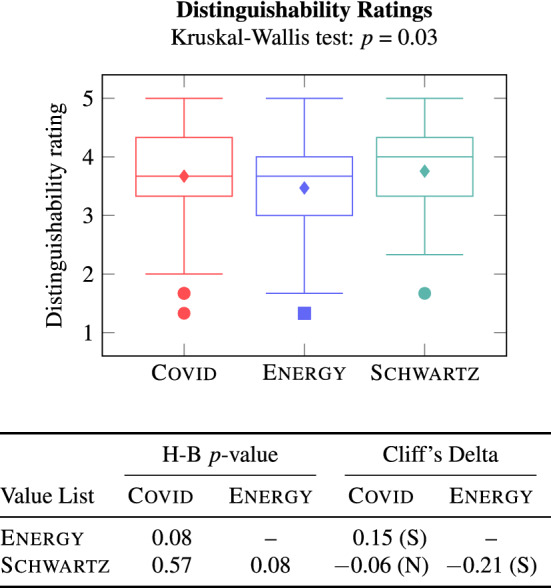
Distinguishability ratings of Axies and Schwartz values

For each value pair in a value list, three crowd workers indicated how distinguishable the values in the pair were. Figure 8 shows the mean distinguishability ratings for pairs of values in the Covid, Energy, and Schwartz value lists.

We notice that the distinguishability of value pairs in Axies and Schwartz lists is not significantly different. Further, none of the value pairs have the mean distinguishability rating of 1. That is, no two values in any of the value lists are rated as indistinguishable. However, a good number of Axies value pairs—14.3% Covid value pairs and 22.5% Energy value pairs—have a mean distinguishability rating in (1, 3). Thus, although distinguishable, the Axies values within a context have similarities among them. This observation aligns with Schwartz’s [1] postulate that values form a continuum of related motivations. In fact, the mean distinguishability rating of a good number (11.1%) of Schwartz value pairs is also in (1, 3). As expected, values that are adjacent in the Schwartz circumplex received low distinguishability scores (such as Conformity and Tradition, rated 1.67), and values at opposite ends of the circumplex received high scores (such as Self-Direction and Conformity, rated 5).

### Consistency

To evaluate the consistency between the two value lists for the same context, we employ the crowdsourced opinion annotations. Recall (from Sect. 4.3.3) that each of the 100 opinions selected for each context was annotated by three crowd workers with the Axies value lists generated for that context. We consider an opinion *o* as annotated with a value *v* if at least two of the three annotations for *o* include *v*.

Let $v_1 \in$ Covid-G1 and $v_2 \in$ Covid-G2, and $O_1$ and $O_2$ be the set of opinions annotated with $v_1$ and $v_2$, respectively. Then, we measure the association between the two values as the Jaccard similarity between their opinion annotations:

2 $$\begin{aligned} J(v_1, v_2) = \frac{|O_1 \cap O_2|}{|O_1 \cup O_2|} \end{aligned}$$

**Fig. 9:**
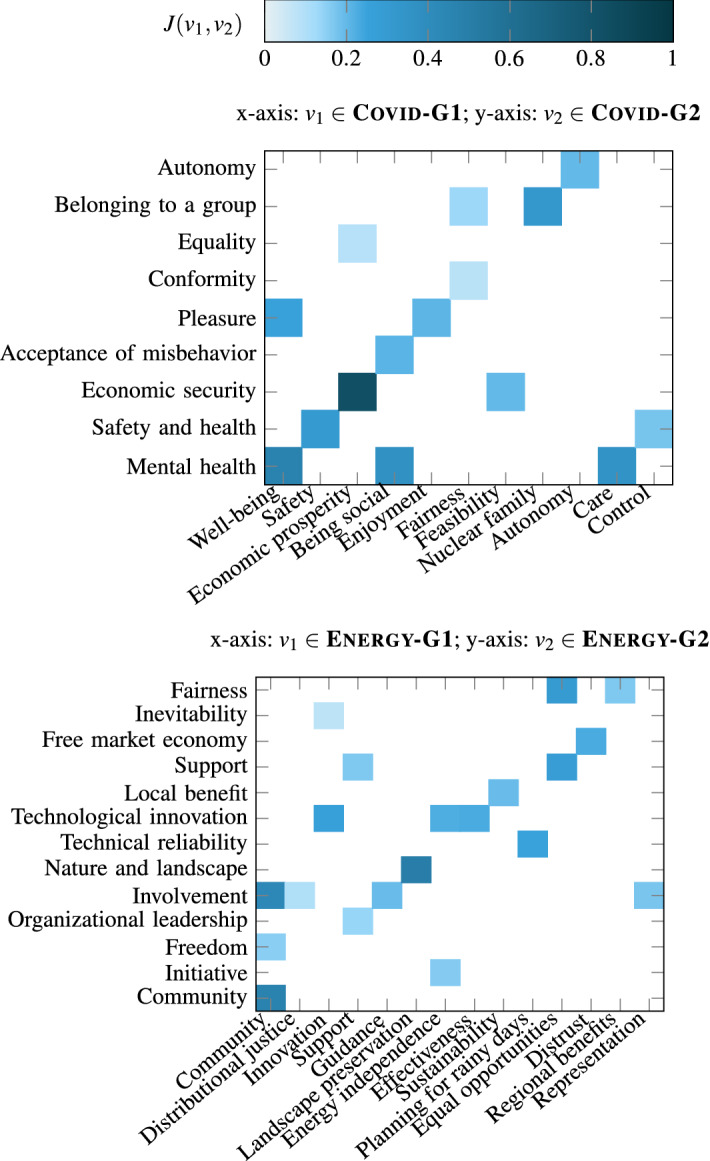
Association between G1 and G2 value lists

For each value in one value list for a context, Fig. 9 shows the closest value in the other list for the context, to emphasize the associations between the two lists.

Although value lists for the same context are not identical, we observe that each value in one list for a context is associated (has a non-zero Jaccard similarity) with at least one value in the other list for that context. In some cases, the association is apparent from the value names, e.g., Economic prosperity $\in$ Covid-G1 and Economic security $\in$ Covid-G2. In some cases, despite differences in the names, the values capture similar motivations, e.g., Planning for rainy days $\in$ Energy-G1 and Technical reliability $\in$ Energy-G2, capture the same motivational goal of planning for unforeseen circumstances. In some cases, the motivation behind a value in a list was distributed over more than one value in the other list. For example, Fairness $\in$ Energy-G2 is captured by Equal opportunities and Regional benefits $\in$ Energy-G1. In essence, no value is conceptually exclusive to one value list within a context.

### Relationship

**Fig. 10:**
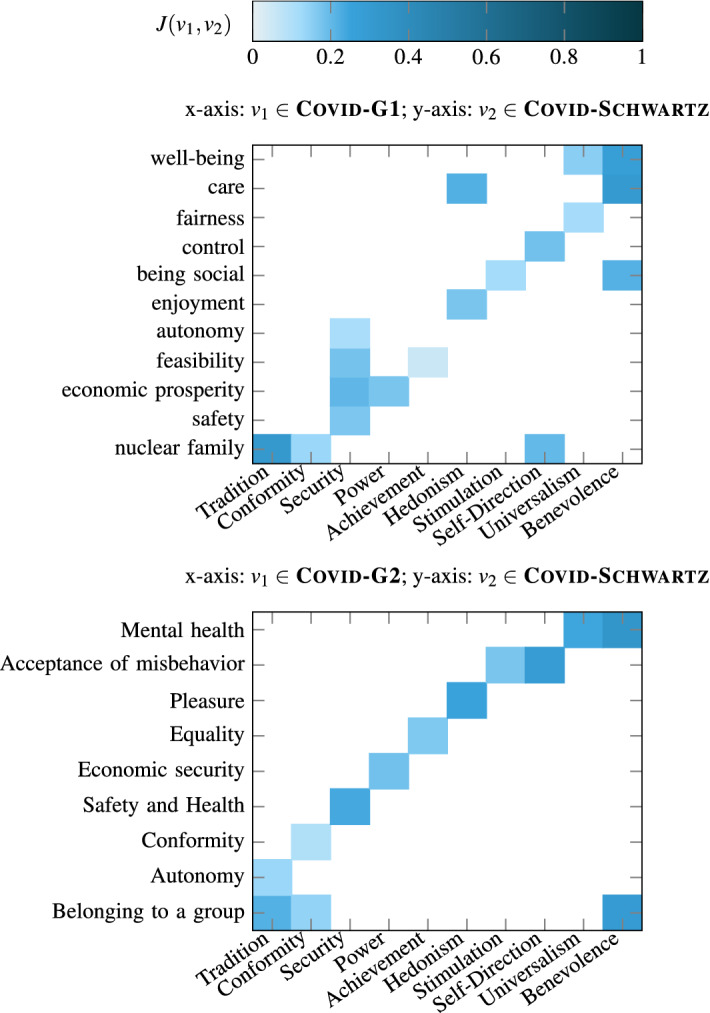
Association between Axies and Schwartz values in the Covid context

**Fig. 11:**
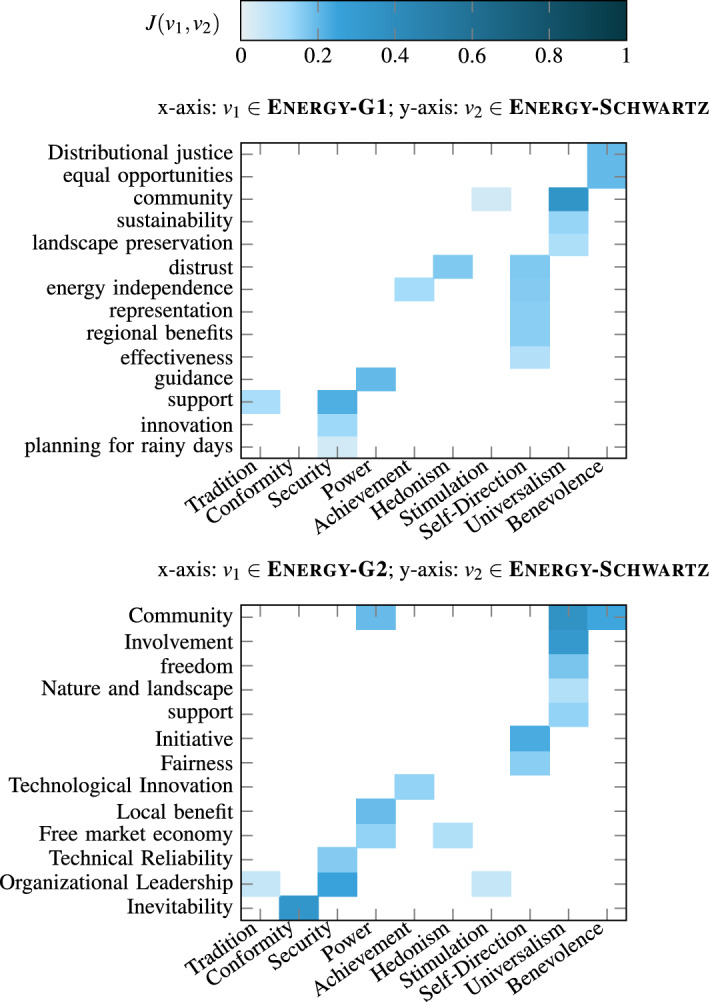
Association between Axies and Schwartz values in the Energy context

Recall that, similar to Axies value annotations, each of the 100 opinions selected for each context was also annotated by three annotators with the Schwartz value list, resulting in the Covid-Schwartz and Energy-Schwartz annotations. To investigate the relationship between Axies and Schwartz value lists, we employ an approach similar to the consistency evaluation (Sect. 5.4). That is, based on the annotations on the same set of opinions, we compute the Jaccard similarity between two values in different value lists as depicted in Figs. 10 and 11.

First, we observe that, each Schwartz value has an association (non-zero Jaccard similarity) with at least one Axies value in each of the four Axies value lists, except for the Schwartz value of Conformity which has no association in the Energy-G2 list. However, the intensity of association is low, overall. For instance, the Schwartz values of Achievement and Conformity in the Covid context, and Stimulation and Tradition in the Energy context have negligible association with values in both Axies lists generated for those respective contexts.

Second, we notice that some Schwartz values have one-to-many relationships with Axies values. This can be clearly observed in the Energy context, where Schwartz values such as Self-Direction and Universalism have multiple matches with both Axies lists. The expected behavior can be also partly observed in the relationship between Covid-G1 and Schwartz value lists (e.g., Security and Benevolence). However, it is less evident in the comparison between Covid-G2 and Schwartz values, where it can only be partially noticed (e.g., Benevolence).

The results above suggest that the relationship between Schwartz and Axies values depends on the context for which the Axies values are generated. In our case, since Energy is a specialized context, only a few general Schwartz values have clear and multiple associations with the context-specific Axies values. In contrast, since the Covid context covers many aspects of life, the Axies values generated for this context have more association with the general Schwartz values.

### Application

**Fig. 12:**
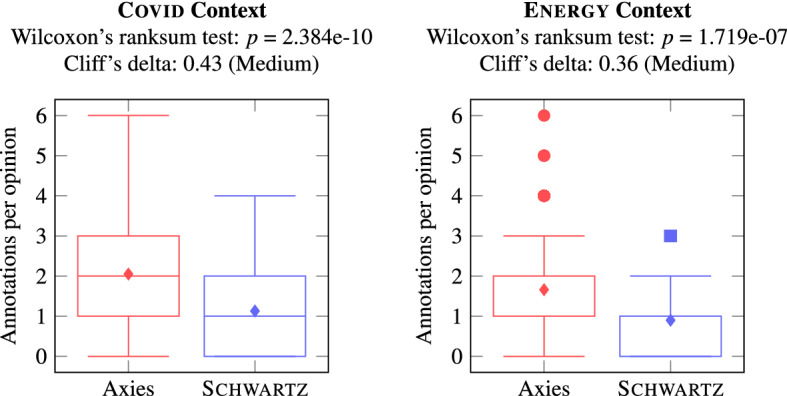
Number of annotations with values belonging to a value list

To assess the application of the value lists, we analyze the opinion annotations. Figure 12 shows the number of annotations per opinion with Axies and Schwartz value lists. In both contexts, the Axies values were annotated significantly more often than the Schwartz values. This suggests that the Axies values are easier to recognize than the Schwartz values in the opinions collected in a context.

**Fig. 13:**
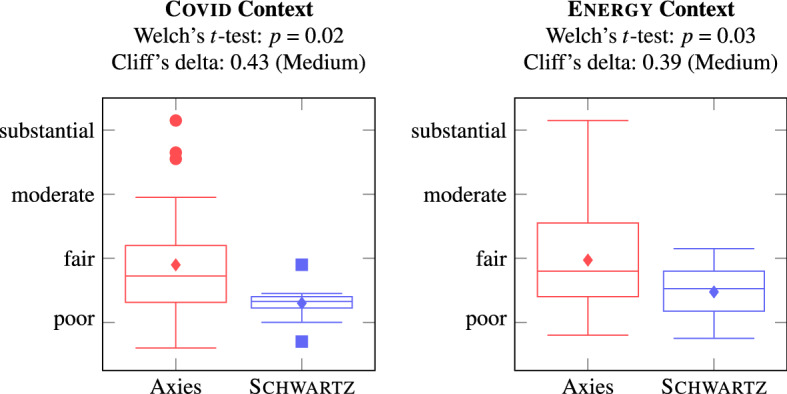
Inter-Rater Reliability of annotations with Axies and Schwartz values

Subsequently, we compare the Inter-Rater Reliability (IRR), measured via Fleiss’ Kappa, of the annotations with the value lists. Figure 13 presents the aggregated IRR [68] for Axies and Schwartz values (Appendix B.2.3 includes IRR for each value).

The IRR is significantly higher for Axies values compared to Schwartz values in both contexts. The average agreement with the Schwartz values is poor, with only two values reaching a fair agreement. In contrast, a large number Axies values is annotated with a fair agreement and some Axies values reach substantial agreement. This suggests that the annotators interpret Axies values more consistently than the (general) Schwartz values, which is desirable in concrete applications of values.

The IRR is low for all value lists, which can be attributed to the inherent difficulty of annotating values [73], especially for untrained crowd workers. Further, some values were annotated only a few times, rendering the agreement difficult to evaluate.

### Threats to validity

We identify three main types of threats to the validity of our findings according to the classification by Cook and Campbell [74].

**Conclusion validity** concerns the ability to draw correct conclusions from the outcome of an experiment. To answer the RQs on the specificity, comprehensibility, and application of value list, we employ rigorous statistical methods, validating the underlying assumptions (e.g., normality assumption for *t*-test) and performing necessary post-hoc analyses (e.g., correcting *p*-values during multiple comparisons). Thus, the findings on these RQs are robust. However, we could not perform statistical analyses in answering the RQs on the consistency and relationship between the value lists. Although our qualitative analyses yield valuable insights on these RQs, we recognize that these findings must be validated again via better experiment designs.

**Internal validity** concerns the influences that may affect the independent variables under study with respect to causality. The subjective interpretation of values is a natural threat to validity in all our experiments. For example, the differences we observe among value lists may be influenced by the differences in the value conceptions of the annotators. The Axies methodology seeks to mitigate this threat by including the consolidation phase, where the annotators discuss their differences in interpretation. Further, in our experiments, we employ two groups of annotators and two contexts to reduce the effect of subjectivity.

**External validity** concerns the limits to generalize the results of our experiment. The small number of annotators who performed the Axies methodology and the limited number of contexts under analysis may reduce the generalizability of our conclusions. First, we required the annotators who performed the Axies methodology (as in Experiment 1) and the policy experts who evaluated context-specificity (as in Experiment 2) to be familiar with the concept of values. Our subjects in these experiments met this requirement but they were all highly educated, living in the Netherlands, and aged between 20 and 35. Thus, the effects of a larger difference in the value annotators’ and policy experts’ education, residence, and age on findings on Experiments 1 and 2 remains to be studied. In Experiment 3, we evaluated the features of the values with the help of laypeople, employing a sample of 72 annotators. Although these annotators are from diverse backgrounds (Appendix A.3.1 provides an overview of the annotators’ demographics), the sample of annotators is not representative of the real population, e.g., the majority of the annotators in the sample are from Europe. Thus, additional experiments with a more representative set of annotators are necessary to generalize the results to a larger population. Third, the experiments have shown slight variations of outcomes across different contexts (Sects. 5.2, 5.3, and 5.5). Further experiments on a varied array of contexts would help in determining the generalizability of our findings. Finally, we compare the Axies value lists with only one list of general values, the Schwartz value list. However, there are other lists of general values, such as Gouveia et al. [75], Hofstede [76], and Inglehart [77]. Although there are similarities and differences among these value lists, empirical data on comparisons of general value lists is sparse [65]. Thus, the generalizability of our findings to general value lists other than the Schwartz value list remains to be studied.

## Conclusions and future directions

Axies combines human and artificial intelligence to yield context-specific values. In a specific context, e.g., driving, context-specific values can be more effective in explaining and predicting human behavior than general values [78]. An autonomous driving agent can concretely elicit its passengers’ preferences over driving-specific values (e.g., safety and efficiency) to tailor the driving experience.

Our experiments highlight important properties of Axies and the trade-offs between context-specific and general values. First, Axies yields values that are *comprehensible* (clear and distinct) to the end users. Comprehensibility is important for an agent to (1) elicit value preferences from users, e.g., by asking whether mental health is more important to a user than conformity in a context, and (2) explain that the agent made a certain decision because the agent inferred, e.g., fairness as more important to the user than regional benefits in the decision context. However, based on value annotators’ feedback and crowd distinguishability results, we observe that values in a context have similarities since they form a motivational continuum. An interesting research direction is to identify and visualize a value continuum (e.g., as a circumplex [1]) from a list of context-specific values. We conjecture that such a visualization would support the process of building a cohesive value list.

Second, as a methodology, we expect Axies to yield reproducible results. Following Axies to annotate an opinion corpus should yield *consistent* value lists independent of the annotators. However, considering the subjective judgements involved, we do not expect a value list produced for a context by one group to be identical to the value list produced by another group. As expected, the value lists generated for the same context by different groups of annotators are not identical but consistent in that each value in one list is associated with one or more values in the other list.

Third, a key result from our experiments is that Axies yields *context-specific* values as it set out to. Specifically, we observe that the values identified for a context are more useful for decision making in that context than in another context. However, some context-specific values are more broadly applicable than others.

Fourth, we perform an empirical comparison between the context-specific (Axies) values and general (Schwartz) values. Our results indicate that Axies values are indeed more context-specific, but slightly less clear to laypeople than Schwartz values. However, when put to the concrete *application* of value annotation, the same laypeople annotate Axies values more often and with higher agreement. This illustrates the suitability of context-specific values for practical applications.

Finally, we explore the *relationship* between Axies and Schwartz values. Our results show that only a few Schwartz values have a clear correspondence to Axies values (i.e., only the Schwartz values that are relevant to the context), and that values with a clear correspondence are often related to multiple Axies values that describe them in a more fine-grained manner in the context. However, we suggest performing more extensive experiments to validate these findings on a varied set of contexts.

Identifying context-specific values is a significant effort. Axies simplifies this process and systematically guides the annotators, who need not be design experts. An interesting future direction is to analyze the benefits of NLP and active learning on the overall process (e.g., by comparing Axies to a baseline without the AI components). Further, in our experiments, the annotators followed the Axies steps one time. In practice, Axies can be used in an agile manner with multiple exploration-consolidation sprints with feedback from evaluations in between the sprints.

Axies starts with the assumption that the context for which values are to be identified is already defined. However, defining a context, in itself, is a significant challenge and an essential step in engineering ethical agents [79]. A context may incorporate a variety of spatio-temporal and social elements that influence the interactions among users and agents [80]. Thus, it is important that the opinion corpus Axies employs is representative of the intended context. For example, in our experiments, the Covid corpus contains the opinions of the residents of a country. Thus, the resulting values are applicable to the residents, but they may not be adequate to capture the values of the healthcare providers (another stakeholder group; thus, a different context). An interesting direction is to employ Axies to compare and contrast contexts. That is, given the Axies value lists for two contexts, the differences between the values in the two lists may indicate the differences between the two contexts.

Value alignment is a long-term research priority for beneficial and robust AI [5]. Our research supports a crucial step in the creation of value-aligned artificial agents—the identification of the values that an agent ought to align with. The values identified via our method can serve as the vocabulary for addressing additional challenges of value alignment such as the translation of values into norms and behaviors [51] and the verification of value adherence to norms [30]. To this end, a repository of values where values are linked with contexts and opinions would be valuable. Given such a repository, designers and developers can reuse values suitable for their contexts and an agent can automatically pick relevant values for a decision context.

## Electronic supplementary material

Below is the link to the electronic supplementary material.

Electronic supplementary material 1 (PDF 171 kb)

Electronic supplementary material 2 (PDF 631 kb)

Electronic supplementary material 3 (PDF 94 kb)

Electronic supplementary material 4 (PDF 77 kb)

## Footnotes

1 www.prolific.co.

## References

[1] Schwartz SH (2012). An overview of the Schwartz theory of basic values. Online Readings in Psychology and Culture.

[2] Murukannaiah, P. K., Ajmeri, N., Jonker, C. J. M., & Singh, M. P. (2020) . New foundations of ethical multiagent systems. In *Proceedings of the 19th international conference on autonomous agents and multiagent systems, AAMAS ’20. Aukland, New Zealand, IFMAAMAS*, (pp. 1706–1710).

[3] Akata Z, Balliet D, de Rijke M, Dignum F, Dignum V, Eiben G, Fokkens A, Grossi D, Hindriks K, Hoos H, Hung H, Jonker CJM, Monz C, Neerincx M, Oliehoek F, Prakken H, Schlobach S, van der Gaag L, van Harmelen F (2020). A research agenda for hybrid intelligence: Augmenting human intellect with collaborative, adaptive, responsible, and explainable artificial intelligence. Computer.

[4] Gabriel I (2020). Artificial intelligence, values, and alignment. Minds and Machines.

[5] Russell S, Dewey D, Tegmark M (2015). Research priorities for robust and beneficial artificial intelligence. AI Magazine.

[6] Soares N, Fallenstein B (2017). Agent foundations for aligning machine intelligence with human interests: A technical research agenda. The technological singularity: Managing the journey.

[7] Balakrishnan, A., Bouneffouf, D., Mattei, N., & Rossi, F. (2019) . Incorporating behavioral constraints in online ai systems. In *Proceedings of the thirty-third AAAI conference on artificial intelligence , AAAI ’19, Honolulu, Hawaii, USA,* (pp. 3–11). AAAI Press. 10.1609/aaai.v33i01.33013.

[8] Soares, N. (2014). *The value learning problem. Technical report, Machine Intelligence Research Institute, Berkeley, California, USA*.

[9] Ajmeri, N., Guo, H., Murukannaiah, P. K., & Singh, M. P. (2020) . Elessar: Ethics in norm-aware agents. In *Proceedings of the 19th international conference on autonomous agents and multiagent systems, AAMAS ’20, Auckland, New Zealand,* (pp. 16–24). IFAAMAS.

[10] Conitzer, V., Sinnott-Armstrong, W., Borg, J. S.,Deng, Y., & Kramer, M. (2017) . Moral decision making frameworks for artificial intelligence. In *Proceedings of the thirty-first AAAI conference on artificial intelligence, AAAI ’17, San Francisco, California, USA, * (pp. 4831–4835). AAAI Press.

[11] Cranefield, S., Winikoff, M., Dignum, V., & Dignum, F. (2017) . No pizza for you: Value-based plan selection in bdi agents. In *Proceedings of the Twenty-Sixth International Joint Conference on Artificial Intelligence, IJCAI ’17, Melbourne, Australia, * (pp. 178–184). International Joint Conferences on Artificial Intelligence Organization. 10.24963/ijcai.2017/26.

[12] Mercuur R, Dignum V, Jonker CM (2019). The value of values and norms in social simulation. Journal of Artificial Societies and Social Simulation.

[13] Rokeach M (1973). The nature of human values.

[14] Graham J, Haidt J, Nosek BA (2009). Liberals and conservatives rely on different sets of moral foundations. Journal of Personality and Social Psychology.

[15] Friedman, B., Kahn, P. H., & Borning, A. (2008) . Value sensitive design and information systems. In *The handbook of information and computer ethics*, (pp. 69–101). Wiley. 10.1002/9780470281819.ch4.

[16] Wilson, S. R., Shen, Y., & Mihalcea, R. (2018) . Building and validating hierarchical lexicons with a case study on personal values. In *Proceedings of the 10th international conference on social informatics, SocInfo ’18, St. Petersburg, Russia, * (pp. 455–470). Springer.

[17] Le Dantec, C. A., Poole, E. S., & Wyche, S. P. (2009) . Values as lived experience. In *Proceedings of the 27th international conference on Human factors in computing systems, CHI ’09, New York, USA, * (p. 1141). ACM Press. 10.1145/1518701.1518875.

[18] Pommeranz A, Detweiler C, Wiggers P, Jonker CM (2012). Elicitation of situated values: Need for tools to help stakeholders and designers to reflect and communicate. Ethics and Information Technology.

[19] de Wet J, Wetzelhütter D, Bacher J (2018). Revisiting the trans-situationality of values in Schwartz’s Portrait Values Questionnaire. Quality and Quantity.

[20] Solove DJ (2006). A taxonomy of privacy. University of Pennsylvania Law Review.

[21] Datler G, Jagodzinski W, Schmidt P (2013). Two theories on the test bench: Internal and external validity of the theories of Ronald Inglehart and Shalom Schwartz. Social Science Research.

[22] van Raaij WF, Verhallen TMM (1994). Domain-specific market segmentation. European Journal of Marketing.

[23] van de Poel, I. (2013) . Translating values into design requirements. In *Philosophy and engineering: Reflections on practice, principles and process* (pp. 253–266). Springer. 10.1007/978-94-007-7762-0_20

[24] Murukannaiah, P. K., & Singh, M. P. (2014) . Xipho: Extending tropos to engineer context-aware personal agents. In *Proceedings of the 13th international conference on autonomous agents and multiagent systems, AAMAS ’14, Paris, France, * (pp. 309–316). IFAAMAS.

[25] Montes, N., & Sierra, C. (2021) . Value-Guided Synthesis of Parametric Normative Systems. In *Proceedigs of the 20th international conference on autonomous agents and multiagent systems, AAMAS ’21, * (pp. 907–915). IFAAMAS.

[26] Serramia, M., Lopez-Sanchez, M., & Rodriguez-Aguilar, J. A. (2020) . A qualitative approach to composing value-aligned norm systems. In *Proceedings of the 19th international conference on autonomous agents and multiagent systems, AAMAS ’20, Auckland, New Zealand, * (pp. 1233–1241) IFAAMAS.

[27] Tielman, M. L., Jonker, C. M., & Van Riemsdijk, M. B. (2019) . Deriving norms from actions, values, and context. In *Proceedings of the international joint conference on autonomous agents and multiagent systems, AAMAS ’19, * (pp. 2223–2225)

[28] Chhogyal, K., Nayak, A., Ghose, A., & Dam, H. K. (2019) . A Value-based Trust Assessment Model for Multi-agent Systems. In *International joint conference on artificial intelligence, IJCAI ’19, * (pp. 194–200). 10.24963/ijcai.2019/28.

[29] Mehrotra, S., Jonker, C. M., & Tielman, M. L. (2021) . More similar values, more trust?—The effect of value similarity on trust in human-agent interaction. In *Proceedings of the 2021 AAAI/ACM conference on AI, ethics, and society, AIES ’21, * (pp. 1–7). Association for Computing Machinery, 10.1145/3461702.3462576.

[30] Tubella, A. A., Theodorou, A., Dignum, F., & Dignum, V. (2019) . Governance by glass-box: Implementing transparent moral bounds for AI behaviour. *IJCAI International Joint Conference on Artificial Intelligence*, August:5787–5793, 10.24963/ijcai.2019/802.

[31] Mouter N, Hernandez JI, Itten AV (2021). Public participation in crisis policymaking. How 30,000 Dutch citizens advised their government on relaxing COVID-19 lockdown measures. PLoS ONE.

[32] Glaser BG, Strauss AL (1967). The discovery of grounded theory.

[33] Basu, S., Banerjee, A., & Mooney, R. J. (2004) . Active semi-supervision for pairwise constrained clustering. In *Proceedings of the 2004 SIAM International Conference on Data Mining, SDM ’04, Orlando, Florida, USA,* (pp. 333–344). Society for Industrial and Applied Mathematics. 10.1137/1.9781611972740.31.

[34] Liscio, E., van der Meer, M., Siebert, L. C., Jonker, C. M., Mouter, N., & Murukannaiah, P. K. (2021). Axies: Identifying and evaluating context-specific values. In *Proc. of the 20th international conference on autonomous agents and multiagent systems, AAMAS ’21* (pp. 799–808). Online, IFAAMAS.

[35] Liscio, E., van der Meer, M., Siebert, L. C., Jonker, C. M., Mouter, N., & P. Murukannaiah. (2021). Axies: Identifying and evaluating context specific values—supplemental material.

[36] Liscio, E., van der Meer, M., Jonker, C. M., & Murukannaiah, P. K. (2021). A collaborative platform for identifying context-specific values. In *Proc. of the 20th international conference on autonomous agents and multiagent systems, AAMAS '21* (pp. 1773–1775). IFAAMAS.

[37] Mooijman M, Hoover J, Lin Y, Ji H, Dehghani M (2018). Moralization in social networks and the emergence of violence during protests. Nature Human Behaviour.

[38] Liu H, Huang Y, Wang Z, Liu K, Hu X, Wang W (2019). Personality or value: A comparative study of psychographic segmentation based on an online review enhanced recommender system. Applied Sciences.

[39] Lin, Y., Hoover, J., Portillo-Wightman, G., Park, C., Dehghani, M., & Ji, H. (2018) . Acquiring background knowledge to improve moral value prediction. In *Proceedings of the 2018 IEEE/ACM international conference on advances in social networks analysis and mining, ASONAM ’18, * (pp. 552–559). IEEE. 10.1109/ASONAM.2018.8508244.

[40] Hoover J, Johnson K, Boghrati R, Graham J, Dehghani M (2018). Moral framing and charitable donation: Integrating exploratory social media analyses and confirmatory experimentation. Collabra: Psychology.

[41] Garten J, Hoover J, Johnson KM, Boghrati R, Iskiwitch C, Dehghani M (2018). Dictionaries and distributions: Combining expert knowledge and large scale textual data content analysis: Distributed dictionary representation. Behavior Research Methods.

[42] Araque O, Gatti L, Kalimeri K (2020). MoralStrength: Exploiting a moral lexicon and embedding similarity for moral foundations prediction. Knowledge-Based Systems.

[43] Hopp FR, Fisher JT, Cornell D, Huskey R, Weber R (2020). The extended moral foundations dictionary (eMFD): Development and applications of a crowd-sourced approach to extracting moral intuitions from text. Behavior Research Methods.

[44] Ponizovskiy V, Ardag M, Grigoryan L, Boyd R, Dobewall H, Holtz P (2020). Development and validation of the personal values dictionary: A theory-driven tool for investigating references to basic human values in text. European Journal of Personality.

[45] Boyd, R. L., Wilson, S. R., Pennebaker, J. W., Kosinski, M., Stillwell, D. J., & Mihalcea, R. (2015) . Values in words: Using language to evaluate and understand personal values. In *Proceedings of the 9th international conference on web and social media, ICWSM ’15, Oxford, UK,* (pp. 31–40). AAAI Press.

[46] Teernstra, L., van der Putten, P., Noordegraaf-Eelens, L., & Verbeek, F. (2016) . The morality machine: Tracking moral values in tweets. In *Advances in intelligent data analysis XV: 15th international symposium, IDA ’16, Stockholm, Sweden, * (pp. 26–37). Springer.

[47] Mosca, F., & Such, J. M. (2021) . ELVIRA: An explainable agent for value and utility-driven multiuser privacy. In *Proc. of the 20th international conference on autonomous agents and multiagent systems, AAMAS ’21, * (pp. 916–924). IFAAMAS.

[48] Nathan, L. P., Klasnja, P. V., & Friedman, B. (2007) . Value scenarios: A technique for envisioning systemic effects of new technologies. In *CHI ’07 extended abstracts on human factors in computing systems* (pp. 2585–2590). 10.1145/1240866.1241046.

[49] Miller, J. K., Friedman, B., Jancke, G., & Gill, B. (2007) . Value tensions in design: The value sensitive design, development, and appropriation of a corporation’s groupware system. In *Proceedings of the international ACM conference on supporting group work, GROUP, * (pp. 281–290). 10.1145/1316624.1316668.

[50] Friedman, B., & Hendry, D. G. (2012) . The envisioning cards: A toolkit for catalyzing humanistic and technical imaginations. In *Proceedings of the SIGCHI conference on human factors in computing systems* (pp. 1145–1148). 10.1145/2207676.2208562.

[51] Serramia M, López-Sánchez M, Moretti S, Rodríguez-Aguilar JA (2021). On the dominant set selection problem and its application to value alignment. Autonomous Agents and Multi-Agent Systems.

[52] Aldewereld H, Dignum V, Tan Y-H (2015). Design for values in software development.

[53] Ferrario, M. A., Simm, W., Forshaw, S., Gradinar, A., Smith, M. T., & Smith, I. (2016) . Values-first SE: Research principles in practice. In *Proceedings of the 38th international conference on software engineering* (pp. 553–562). 10.1145/2889160.2889219.

[54] Mougouei, D., Perera, H., Hussain, W., Shams, R., & Whittle, J. (2018) . Operationalizing human values in software: A research roadmap. In *ESEC/FSE 2018—Proceedings of the 2018 26th ACM joint meeting on European software engineering conference and symposium on the foundations of software engineering* (pp. 780–784). 10.1145/3236024.3264843.

[55] Perera, H., Mussbacher, G., Hussain, W., Ara Shams, R., Nurwidyantoro, A., & Whittle, J. (2020) Continual human value analysis in software development: A goal model based approach. In *Proceedings of the IEEE international conference on requirements engineering* (pp. 192–203). 10.1109/RE48521.2020.00030.

[56] Detweiler C, Harbers M (2014). Value stories: Putting human values into requirements engineering. CEUR Workshop Proceedings.

[57] Thew S, Sutcliffe A (2018). Value-based requirements engineering: Method and experience. Requirements Engineering.

[58] Winter, E., Forshaw, S., & Ferrario, M. A. (2018) . Measuring human values in software engineering. In *Proceedings of the 12th ACM/IEEE international symposium on empirical software engineering and measurement* (pp. 10–13). 10.1145/3239235.3267427.

[59] Perera, H., Hussain, W., Whittle, J., Nurwidyantoro, A., Mougouei, D., Shams, R. A., & Oliver, G. (2015) . A study on the prevalence of human values in software engineering publications, 2015–2018. In *Proceedings of the 42nd international conference on software engineering* (pp. 409–420). 10.1145/3377811.3380393.

[60] Rescher N (1969). Introduction to value theory.

[61] Reimers, N., & Gurevych, I. Sentence-BERT: Sentence embeddings using siamese BERT-networks. In *Proceedings of the 2019 conference on empirical methods in natural language processing and the 9th international joint conference on natural language processing, EMNLP-IJCNLP ’19, Hong Kong, China, * (pp. 3973–3983). Association for Computational Linguistics. 10.18653/v1/d19-1410.

[62] Rosenkrantz DJ, Stearns RE, Lewis PM (1977). An analysis of several heuristics for the traveling salesman problem. SIAM Journal on Computing.

[63] Mrkšić, N., Séaghdha, D., Thomson, B., Gašić, M., Rojas-Barahona, L., Su, P. H., Vandyke, D., Wen, T. H., & Young, S. (2016) . Counter-fitting word vectors to linguistic constraints. In *Proceedings of the 2016 conference of the North American chapter of the association for computational linguistics: Human language technologies, NAACL HLT ’16, San Diego, California, USA, * (pp. 142–148). Association for Computational Linguistics. 10.18653/v1/n16-1018.

[64] Saunders B, Sim J, Kingstone T, Baker S, Waterfield J, Bartlam B, Burroughs H, Jinks C (2018). Saturation in qualitative research: Exploring its conceptualization and operationalization. Quality and Quantity.

[65] Hanel PH, Litzellachner LF, Maio GR (2018). An empirical comparison of human value models. Frontiers in Psychology.

[66] Spruit, S. L., & Mouter, N. (2020) . 1376 residents of Súdwest-Fryslân about the future energy policy of their municipality: The results of a consultation, 2020. https://www.tudelft.nl/en/tpm/pve/case-studies/energy-in-sudwest-fryslan/.

[67] Junczys-Dowmunt, M., Grundkiewicz, R., Dwojak, T., Hoang, H., Heafield, K., Neckermann, T., Seide, F., Germann, U., Aji, A. F., Bogoychev, N., Martins, A. F. T., & Birch, A. (2018) . Marian: Fast neural machine translation in C++. In *Proceedings of ACL 2018, system demonstrations, ACL ’18, Melbourne, Australia* (pp. 116–121). Association for Computational Linguistics. 10.18653/v1/P18-4020.

[68] Hallgren KA (2012). Computing inter-rater reliability for observational data: An overview and tutorial. Tutorials in Quantitative Methods for Psychology.

[69] Hollander M, Wolfe DA (1999). Nonparametric statistical methods.

[70] Delacre M, Lakens D, Leys C (2017). Why psychologists should by default use Welch’s t-Test instead of Student’s t-Test. International Review of Social Psychology.

[71] Dunn OJ (1964). Multiple Comparisons Using Rank Sums. Technometrics.

[72] Cliff N (2014). Ordinal methods for behavioral data analysis.

[73] Hoover J, Portillo-Wightman G, Yeh L, Havaldar S, Davani AM, Lin Y, Kennedy B, Atari M, Kamel Z, Mendlen M, Moreno G, Park C, Chang TE, Chin J, Leong C, Leung JY, Mirinjian A, Dehghani M (2020). Moral foundations twitter corpus: A collection of 35k tweets annotated for moral sentiment. Social Psychological and Personality Science.

[74] Cook T, Campbell D (1979). Quasi-experimentation—design and analysis issues for field settings.

[75] Gouveia VV, Milfont TL, Guerra VM (2014). Functional theory of human values: Testing its content and structure hypotheses. Personality and Individual Differences.

[76] Hofstede G (2011). Dimensionalizing cultures: The Hofstede model in context. Online Readings in Psychology and Culture.

[77] Inglehart R (1997). Modernization and postmodernization in 43 societies. Modernization and postmodernization.

[78] van den Berg TG, Kroesen M, Chorus CG (2020). Does morality predict aggressive driving? A conceptual analysis and exploratory empirical investigation. Transportation Research Part F: Traffic Psychology and Behaviour.

[79] Ajmeri N, Guo H, Murukannaiah PK, Singh MP (2018). Designing ethical personal agents. IEEE Internet Computing.

[80] Ajmeri, N., Guo, H., Murukannaiah, P. K., & Singh, M. P. (2018) . Robust norm emergence by revealing and reasoning about context: Socially intelligent agents for enhancing privacy. In *Proceedings of the 27th international joint conference on artificial intelligence, IJCAI ’18, Stockholm* (pp. 28–34).

