# Supplementary material for: What values should an agent align with? An empirical comparison of general and context-specific values
Source: Auton Agent Multi Agent Syst. 2022 Mar 28;36(1):23. doi: 10.1007/s10458-022-09550-0 (PMC8958938; doi:10.1007/s10458-022-09550-0)
Supplement: Supplementary file 1 — Electronic supplementary material 1 (PDF 171 kb) [file 10458_2022_9550_MOESM1_ESM.pdf]

A Experiments Protocol

We provide additional information on how the three experiments outlines in Section 4 were conducted.

A.1 Experiment 1: Value Lists

As Section 4.1 describes, six annotators were invited to generate value lists with the use of Axies. A brief survey revealed that the annotators consisted of one graduate student, three doctoral students, and two postdoctoral researchers, aged between 20 and 35, and with previous experience with personal values.

24h before each of their participation to the first exploration, we sent an email to each participant asking to create a user on the web application and accept the Informed Consent Form. Upon acceptance, they were shown general information about personal values and the analyzed contexts (COVID and ENERGY). At the beginning of the first exploration and first consolidation sessions, each annotator was shown instructions and goals of the respective phases. Informed Consent Form, introductory information, and phases instructions are detailed in `Instructions_for_Axies.pdf`.

A.2 Experiment 2: Specificity

As described in Section 4.2, two policy experts were invited to perform Experiment 2. Upon giving informed consent, the experts reported to be graduate students in the *technology and policy making field*, with experience with the two participatory value evaluations (PVEs) at the base of the analyzed contexts (COVID and ENERGY) through previous projects. Before starting the individual phase of the experiment, they were provided with instruction for the evaluation task (including information about the contexts). Informed Consent Form and instructions are detailed in `Instructions_for_Specificity_Evaluation.pdf`.

Both annotators individually gave specificity ratings to all 57 values (including all Axies and Schwartz values). Afterwards, they were invited to deliberate about the values for which their ratings differed more than two points on Likert scale (2 values out of 57). Following their discussion, they were offered the option to change the rating for these values, provided they noted down why they changed it. Table A1 presents the Intraclass Correlation (ICC) coefficients illustrating the agreement between annotators, highlighting the differences between Axies and Schwartz values.

Table A1: ICC of context-specificity ratings

	**Axies values**	**Schwartz values**	**All values**
ICC before discussion	0.69 (good)	0.51 (fair)	0.68 (good)
ICC after discussion	0.76 (excellent)	0.51 (fair)	0.74 (good)

A.3 Experiment 3: Comprehension and Consistency

Section 4.3 presents an overview of Experiment 3. We initially opened a pilot annotation task on Prolific for four user, and set the expected completion time to 50 minutes. Results encouraged us to proceed. Although we expected the completion time to be lower, we preferred to keep the expected completion time to 45 minutes to encourage users to spend more effort on the task.

Upon taking the task on Prolific, workers were redirected to the web application hosted on our servers. Here, after accepting the Informed Consent Form, they were given a small introduction to the annotation task and the assigned context (COVID or ENERGY). Then, they were guided sequentially through the three steps (clarity evaluation, distinguishability evaluation, opinion annotation), while being shown instructions at the beginning of the respective step. Informed Consent Form, introductory information, and steps instructions are all detailed in `Instructions_for_Crowd_Evaluation.pdf`.

A.3.1 User demographics

Upon giving informed consent, workers were asked the following demographic information:

- What is your age?
- What gender do you identify as?
- Where is your home located?
- What is the highest degree or level or education you have completed?

Figure A1 presents the aggregated results of the demographics of the 72 users whose submissions were considered in the study.

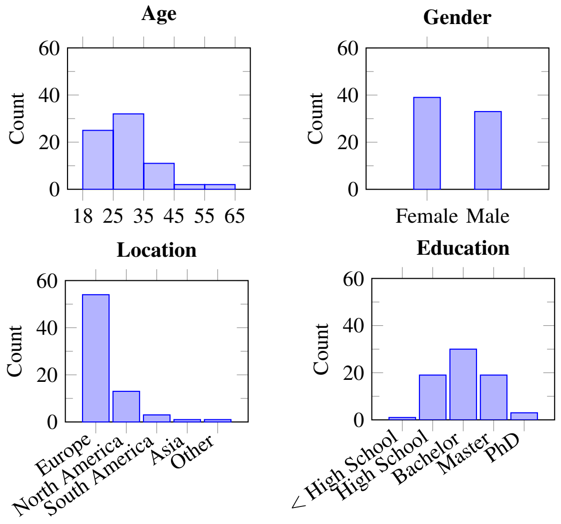

Fig. A1: Demographics of crowd workers

A.3.2 Quality control

As mentioned in Section 4.3.5, four attention checks were included in each task. In distinguishability evaluation, we showed an extra pair composed of twice the same value, which the users were intended to label as not distinguishable (1 out of 5). Then, we showed a pair consisting of two values taken from opposite ends of the Schwartz circumplex (Tradition and Self-Direction), which the users were expected to rate with a distinguishability score of at least 3 out of 5. In the opinion annotation task, each worker was shown two artificial opinions explicitly highlighting one of the values present in the value list (for instance, an opinion such as "Safety comes above all.", with Safety being one of the values present in the list). The check would be considered successfully passed if the related value would be among the values chosen to annotate the opinion.

Workers' submissions were accepted if they would spend at least 20 minutes on the task, or if they would pass more than half of the attention checks. Of the 115 workers who completed the task, 107 fulfilled these requirements and were paid. The submissions were then considered in our results only if both distinguishability evaluation attention checks and at least one of the completeness evaluation attention check (due to the more subjective nature of the latter task) would be successfully passed. 72 submissions were finally considered in our analysis.

B Extended results

We offer additional details on the results presented in Section 5. Raw results are in `raw_results.zip`.

B.1 Value lists

We present a detailed picture of the results obtain with Experiment 1, described in Section 4.1 and 5.1.

B.1.1 Exploration

Six annotators performed exploration on two contexts, resulting in 12 exploration sessions. The main results are presented in Section 5.1.1. An overview of the sessions is presented in Table B1, highlighting the number of values generated in each exploration.

Table B1: Overview of the exploration phase

Annotator ID	Group	Context	#Values	Duration
1	1	COVID	8	55 min
1	1	ENERGY	8	70 min
2	2	COVID	11	80 min
2	2	ENERGY	12	80 min
3	1	COVID	13	60 min
3	1	ENERGY	18	75 min
4	2	COVID	13	80 min
4	2	ENERGY	19	60 min
5	2	COVID	8	60 min
5	2	ENERGY	6	50 min
6	1	COVID	14	80 min
6	1	ENERGY	14	70 min

B.1.2 Consolidation

Two groups (of three annotators each) performed consolidation on two contexts, resulting in four consolidation sessions. The main results are presented in Section 5.1.2. An overview of the sessions is presented in Table B2, indicating the number of values at the start and at the end of each consolidation. The four complete value lists (including value names, keywords, and defining goals) are illustrated in Tables B4, B5, B6, and B7. We retain all keywords as originally annotated, removing one keyword we considered inappropriate.

Table B2: Overview of the consolidation phase

Group	Context	#Start values	#End values	Duration
1	COVID	35	11	105 min
1	ENERGY	40	14	110 min
2	COVID	32	9	115 min
2	ENERGY	37	13	120 min

B.2 Comprehensibility

Here we present a detailed picture of the comprehensibility evaluation results obtain with Experiment 3, described in Section 4.3. The main results are presented in Section 5.3.

B.2.1 Clarity evaluation

Section 5.3.1 presents the clarity evaluation results per each context. Figure B1 presents the average clarity ratings given to the values of the five value lists.

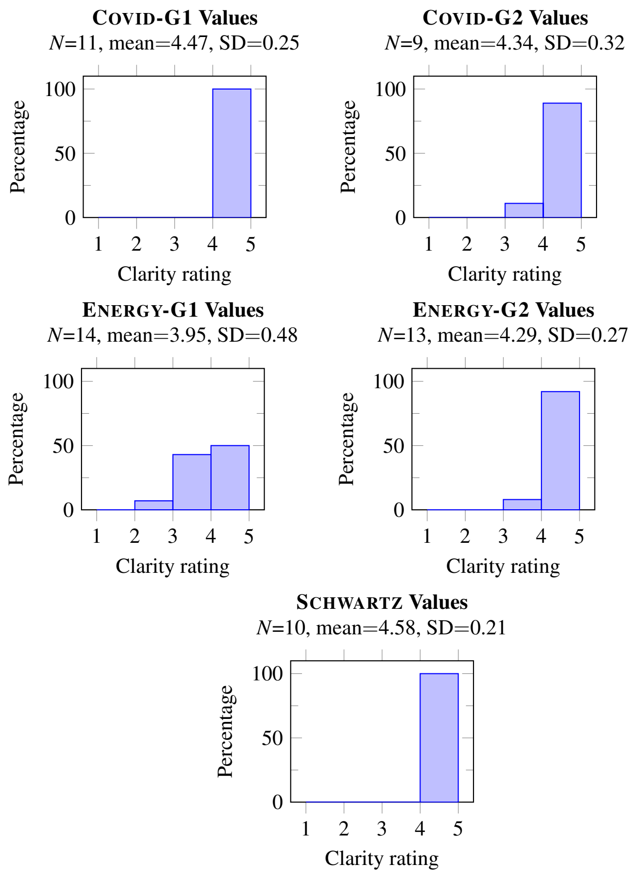

Fig. B1: Histograms of value clarity ratings

B.2.2 Distinguishability evaluation

Section 5.3.2 presents the distinguishability evaluation results per each context. Figure B2 presents the average distinguishability ratings divided in the five value lists.

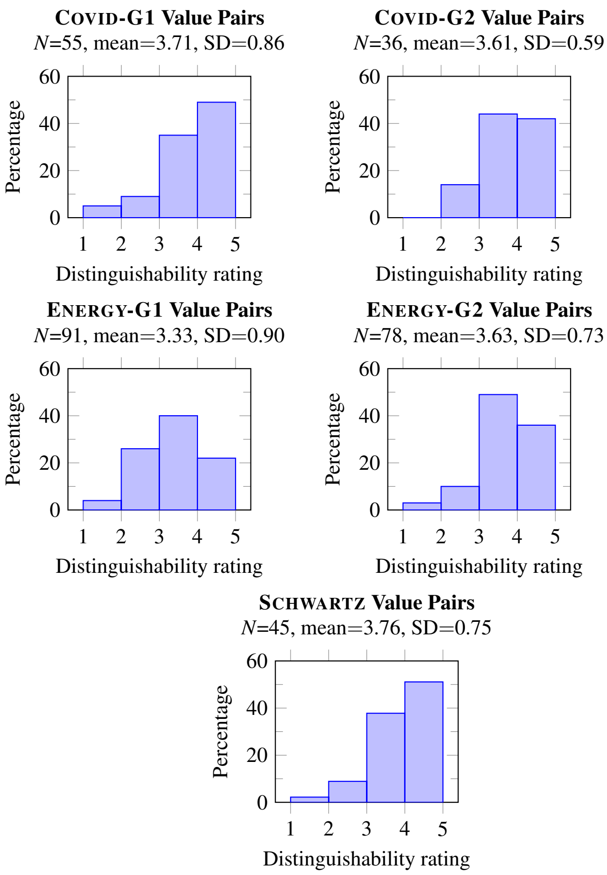

Fig. B2: Histograms of value distinguishability ratings

B.2.3 Crowd Annotation Task

Section 5.6 describes the results of the crowdsourced annotations. Figures B3 and B4 illustrates, per value list, the number of opinions that were annotated with each value belonging to the list. Recall that each value list was used to annotate 100 opinions. Table B3 present the Inter-Rater Reliability (and its interpretation) for each value in the value lists.

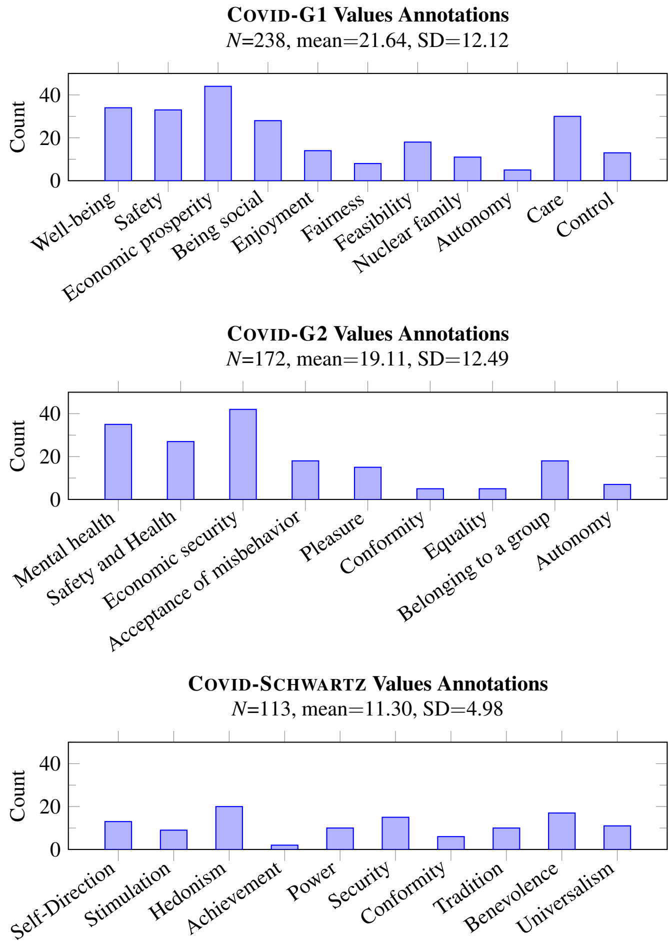

Fig. B3: Histogram of annotated opinions per value in context COVID

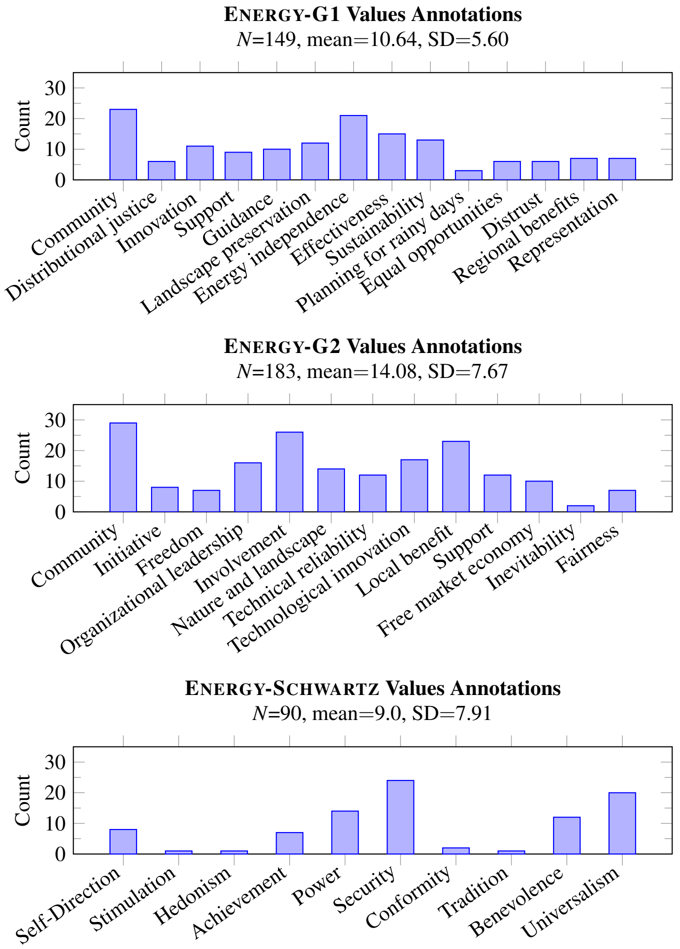

Fig. B4: Histogram of annotated opinions per value in context ENERGY

Table B3: Inter-Rater Reliability with Axies and Schwartz value lists

Covid-G1	
**Value**	**Fleiss' Kappa**
fairness	0.14 (poor)
care	0.1 (poor)
being social	0.22 (fair)
enjoyment	0.1 (poor)
economic prosperity	0.53 (moderate)
nuclear family	0.27 (fair)
control	-0.08 (poor)
safety	0.08 (poor)
autonomy	-0.07 (poor)
well-being	0.15 (poor)
feasibility	-0.06 (poor)

Covid-G2	
**Value**	**Fleiss' Kappa**
Mental health	0.51 (moderate)
Safety and Health	0.23 (fair)
Economic security	0.63 (substantial)
Acceptance of misbehavior	0.39 (fair)
Pleasure	0.22 (fair)
Conformity	-0.02 (poor)
Equality	0.08 (poor)
Belonging to a group	0.16 (poor)
Autonomy	0.01 (poor)

Energy-G1	
**Value**	**Fleiss' Kappa**
Distributional justice	-0.03 (poor)
innovation	0.22 (fair)
guidance	0.1 (poor)
energy independence	0.28 (fair)
effectiveness	0.05 (poor)
support	0.09 (poor)
sustainability	0.03 (poor)
planning for rainy days	0.08 (poor)
distrust	0.27 (fair)
landscape preservation	0.63 (substantial)
equal opportunities	0.15 (poor)
regional benefits	-0.04 (poor)
representation	0.03 (poor)
community	0.41 (moderate)

Energy-G2	
**Value**	**Fleiss' Kappa**
Community	0.31 (fair)
Initiative	0.08 (poor)
freedom	0.34 (fair)
Organizational Leadership	0.26 (fair)
Involvement	0.32 (fair)
Nature and landscape	0.32 (fair)
Technical Reliability	0.16 (poor)
Technological Innovation	0.31 (fair)
Local benefit	0.08 (poor)
support	0.12 (poor)
Free market economy	0.31 (fair)
Inevitability	0.23 (fair)
Fairness	0.14 (poor)

Covid-Schwartz	
**Value**	**Fleiss' Kappa**
Self-Direction	0.06 (poor)
Stimulation	0.06 (poor)
Hedonism	0.07 (poor)
Achievement	0 (poor)
Power	0.04 (poor)
Security	0.08 (poor)
Conformity	-0.06 (poor)
Tradition	0.08 (poor)
Benevolence	0.18 (poor)
Universalism	0.09 (poor)

Energy-Schwartz	
**Value**	**Fleiss' Kappa**
Self-Direction	0.11 (poor)
Stimulation	0.02 (poor)
Hedonism	0.2 (fair)
Achievement	-0.05 (poor)
Power	0.23 (fair)
Security	0.13 (poor)
Conformity	-0.04 (poor)
Tradition	0.08 (poor)
Benevolence	0.17 (poor)
Universalism	0.1 (poor)

Table B4: Complete COVID-G1 value list

Value name	Keywords	Defining goal
fairness	apply to everyone, differences, equality, distribution, discrimination	Measures should apply to everyone, without discriminating among groups (age and region especially).
care	caregiving, care for each others, help, support	Make sure that everyone is taken care of and looked after.
being social	contact, physical, oil of society, social isolation, neighbors, hug, weighs very heavily, fellow believers, religion, friends, each others	Being in contact with friends, neighbors and other acquaintances in your social circle.
enjoyment	beer, getaway, recreational sex, sport, entertainment, drinking, fun, pleasure, celebrate, festivals, weekends	Being able to enjoy life at full and indulge in fun activities.
economic prosperity	unemployment, poverty, bankruptcy, economy, companies, entrepreneur, money, interests, restart, must be kept running, running, income, provide, jobs, industry, work, worried, stability	Being able to pay and afford what you need.
nuclear family	couch, wedding, parents, sister, birth, family members, foundation, social education, unnatural, child, relatives	Being together with your parents, kids and siblings.
control	cross-border traffic, traffic, necessary adjustments, protective equipment, distance, necessary, obliging mouth caps, can maintain, tests, impossible, protectors, solutions, masks, desinfect, app, protocol, monitored, humidity monitored, difficult, distance, sly, secretive, obey	Having and respecting regulations in order to avoid the spread of the disease.
safety	safety measures, mouth caps, gloves, precaution, distance, wash hands, low risk, not transfering, many strangers come very close, immunity temporary, infection hazard, transfer limited, below 60, risk, building immunity, group immunity, tests, dangers to the elderly, monitoring our health, health	Staying healthy and not infected.
autonomy	everything open, restrictions, enforcement, choice, own, liberty, individualist, self-concern	Being able to determine by yourself what you do and are allowed to do.
well-being	alone, loneliness, psychological influence, relaxation, depression, mental health, emotional, beautifull moments, mental health, psychically, suicide, pressure, needs, normal, loneliness, isolation, restless, apprehensiveness, expectation, hope	Being content, doing well, without worries.
feasibility	test case, research, possibilities, demonstrated, charts, statistics, science, rivm, try out, unlikely, logical, practical, reasonable, common sense	Having policies that are doable and effective.

Table B5: Complete COVID-G2 value list

**Value name**	**Keywords**	**Defining goal**
Mental health	mentally, stress, Mental damage, positivity, living alone, psychic injury, stress, physical proximity, mental complaints, tension, spiritual relaxation, quality of life, psychiatry, loneliness, walk around, visit ill people in nursery home, cheer up, last days, empathy, compassion, last phase of life, psychologist, personal care	The strive towards protecting and improving one's emotional and psychological well-being
Safety and Health	hygiene, facial protection, screen, immune, small scale, masks, protected, keep distance, distance, Mouth caps, gloves, busy, crowded, health, groups, personal, safe, mortality, fatality, death, dying, diabets, die, immune system, temperature	Personal protection against the health-related impacts caused by the coronavirus
Economic security	money, shops, recession, company, jobs, entrepreneurs, self-employed, survival, companies, financial impact, fall, restart activity, poverty, bankrupt, restaurants, enterpreneurship, economic, taxation, productivity, unemployment, resume work, heavy economic times, economy, zzp, zzper, financial damage, companies fall down, earn money, Relaunching economics	Mitigating the economic downsides of the situation caused by the pandemic and its countermeasures.
Acceptance of misbehavior	working black, empathy, illegal, ignorance, misbehavior, People are already doing it, We see it every day, abuse, illusion, lying, unfair	Acceptance to the fact that people might not conform to measures.
Pleasure	pleasure, fun, activity, entertainment, cozy, Drink, Balance, social interaction, festivals, dates, food, recreational, leisure, liveliness, kiss, hug	Being able to undertake activities that promotes personal satisfaction and pleasure.
Conformity	official, government, behavior, citizen, control, measures, controlled protection, 1.5 meter, working undercover, RIVM, keep distance, strict regulations, mouth caps, rules, fines, norms, regulations, stay home, responsible, unless you're ill, no busy places, limit risk, work at home, trust	Striving to comply to the guidelines and rules imposed by the authorities
Equality	equal, the same, unfair, Distinction, discrimination, doesn't apply only to certain groups of subjects, help other people, Human side, humanity	Ensuring that all people are given the same treatment and act for the common good
Belonging to a group	friends, miss friends, social contact, Zoom, Skype, meet friends, social, cozy, chill, friends, buddies, youth, together, contact, church, sing, pray, sports, family, school, peers, daughter, cuddling, grandparent, relatives	Being able to closely interact with the people that you care about or enjoy spending time with
Autonomy	walk around, own decision, choice, proximity, do something useful, police state, personal, choice, freedom, self, autonomy, companies decide, decide for themselves, responsibility for individual, you may visit or not, own responsibility, choose for themselves	Being able to make your own decisions and take the responsibility for your actions

Table B6: Complete ENERGY-G1 value list

**Value name**	**Keywords**	**Defining goal**
Distributional justice	Everywhere, not just in Friesland, spread , across the netherlands, they pay	Fair distribution of burdens and benefits.
innovation	alternatives, stimulate, under development, future, bet on, biogas, new technologies, creativity, progress, invention	Keep on producing new and better technologies.
guidance	direction, Obliges companies, control, central, disagree, coordinated, overview, centrally regulated, distribute, take the lead, government, municipality, monitoring	Having a central entity that decides and regulates energy policies.
energy independence	themselves, own backyard, self, self-doing, close to home, private, reserve, need, storage	Having an independent source of energy, without relying on external providers.
effectiveness	success, feasible, effective, appropriate, optimal, in order to get away from , inevitable, necessary, does its job, very busy, well-led, needed, Prevent things from being done twice, Many different housing situations, possible, small, fitting	Creating tailor-made, doable policies to reach the renewable energy target.
support	help, care, possibilities, don't know, knowledge, unable, weakest	People receive advice and assistance.
sustainability	pollution, renewable energy, care for environment	Having energy policies that increase renewable energy generation.
planning for rainy days	expectation, storage, seasonal, weather conditions, unforeseen	Having plans for unforeseen circumstances.
distrust	only revolves around money, fill his own cases, not leave it to the market, small part, delivered to the gods, serving, repugnance, economy, savings, anti-politics, many beautiful words, few deeds, anti-market, mistrust	Big players (government, large companies) should not be in charge of solving problems on citizens' behalf.
landscape preservation	Billiard towel with holes, beautiful, landscape, messy, few places as possible, not stand out, landscape pollution, inconspicuous, opposed to large-scale, beauty, nature is affected, interference, for nature, surroundings	Leave landscape untouched.
equal opportunities	benefits everyone, rich and poor, paid by everyone, strongest win from the weakest, possible for everyone, fairness	Everyone should be given a chance to participate and speak up.
regional benefits	jobs, own gain, profit, investment, opportunities	Bring advantages to job market and economy of South-West Friesland.
representation	Approach all residents, stand up for, accountability, responsibility	Every member of society should be accounted for when taking decisions.
community	decide for themselves, determining people, people determining, free, own steps, willingness, leave it to the people, from the bottom, choice, self-management, independence, autonomy, Local needs, own community, small-scale, own initiative, involvement, with residents, participation, each others, solidarity, engagement	Creation and ownership by and of the community.

Table B7: Complete ENERGY-G2 value list

**Value name**	**Keywords**	**Defining goal**
Community	cooperation, Encouraging residents, ideas, involve, involvement, local binder, limits and conditions of government and residents , mei elkoar, mienskip, think along, each other, care, contribute, balance, protection , everyone, Solidarity	Preserving the feeling of doing it together and taking care of each other
Initiative	Involvement, Do something themselves, empty roofs, local enterpreneur, residents, buying solar panels, self-doing, conscious behaviour, heat pumps, solar panels, enthusiasm, regulate its own energy, opportunities, Encouraging residents, Stimulates, stick behind the door, Initiating	Participants value acting towards their own plans
freedom	own direction, my choice, freedom, independence, autonomy, responsibility for themselves, private, liberty, themselves, voice, residents, small scale	Participants value the ability to freely speak, think or make their own choices in the energy transition.
Organizational Leadership	organized, mess, conflict, central management, delays, decision-making, Prevent things from being done twice, oversee, higher level, cooperation, director, supervises, compliance, regulate, energy co-operations, director, leader, control, supervision, protection, direction, conditions, central point, democratic, Consults, municipality, Approach all residents, decision, coordinate, lead, expertise, nation, municipality, Europe, politicians, official	A single organization is in charge of supervision and organizing the process towards reaching the energy goals.
Involvement	involve people, involve citizens, democratically elected, voting, veto, election, financial participation, think along, participation, public, public evaluation, survey, opinion	People have a say in the process of reaching the energy goals
Nature and landscape	Nature, landscape, few places as possible, preserve, nature conservation, find a place, nature protection, small number of places, disruption of landscape, view in nature, ugly windmills, putting trees, trees, bushes, clustering, cluttering of the landscape, landscape, living, surrounding, scenic, ecology, not stand out, flat building, further from the inhabited world, not in my backyard, underground, minimal nuisance, less burdened, minimize burdening, liveability, noise, in front of my nose	Preserving nature and the aesthetic of the landscape
Technical Reliability	Transport losses, Overproduction, help with industry, severe winter, retention of electricity, spikes, excess generated energy, stored, later use, always electricity, stable grid, control, stability, direction of the municipality, electricity grid, energy security	Ensuring that people can rely on energy solutions and have a stable energy grid without hampering in their way of life
Technological Innovation	Hydrogen storage, Frontrunner, water treatment, biogas, wave energy, most recent products, newest technology, H2	Capacity to come up with new and better solutions to energy-related problems
Local benefit	Investment, Profits, revolves around money, earning model, profitable, rewarding, an extra penny, earn money, local profit, labour, financial risk reduction, no big investors, local labour, jobs for citizens, mercy of wealthy companies	To try and steer the (financial) benefits from a solutions towards one that is best for the participant and its peers.
support	municipality can help me with that, professional expertise, help me decide, older people, elderly, support, help, assist, aid, facilitation, legal, permit, subsidy	Ensuring that all participants can rely on the expertise of the decision makers, and are all assisted during the organizational procedures.
Free market economy	profitable, efficient, lucrative, most profit per area, optimize space, scalability, large scale, companies are effective, company, income, players, capitalist , profit	The belief that a free, self-regulated market economy will result in the best gains for all participants
Inevitability	no choice, energy security, necessity, needed, required, necessary, important, uncertainty , responsibility	The realization that actions need to be taken even if your preferences are not aligned with that action
Fairness	Same playing field, social approach, social, Each province must take a share, weaker, share profit, Divide the burden, strongest wins, neighbourhoods differ, local decision, local solution, Keep it local	The strive towards a proper division of benefits and responsibilities.
