# Supplementary material for: What values should an agent align with? An empirical comparison of general and context-specific values
Source: Auton Agent Multi Agent Syst. 2022 Mar 28;36(1):23. doi: 10.1007/s10458-022-09550-0 (PMC8958938; doi:10.1007/s10458-022-09550-0)
Supplement: Supplementary file 2 — Electronic supplementary material 2 (PDF 631 kb) [file 10458_2022_9550_MOESM2_ESM.pdf]

Informed Consent Form

Purpose of this research study: In this study, we aim at creating a list of context-related personal values, based on input provided by participants to a survey.

What you will do in the study: You will annotate personal values based on written input provided by participants to a survey. Additional details will follow.

Time required: It is dependent on you, as it will be explained in the following instructions. Estimated time is 4 hours, divided in four 1 hour slots.

Risks: There are no risks anticipated in this study. However, in case of doubts or concerns, do not hesitate to contact the researchers.

Benefits: You will not directly benefit from participating in this study.

Privacy and confidentiality: Should you agree to take part, your participation will be completely confidential. All information gathered in the survey will be stored securely in compliance with the standards set by the European Union General Data Protection Regulation (GDPR). No one outside the research group will have access to the data during the research period. Background data will be kept by the research group until the analyses are finalized, at the latest in September 2021. By this date, background data will be pseudonymized. Upon analysis and publication, the pseudonymized information will be made available on open access for other researchers to analyse. This study is approved by the YYY Ethics Committee.

Right to withdraw from the study: Participation in the study is completely voluntary. If at any time you do not wish to continue your participation, you are welcome to withdraw from the survey without penalty.

How to withdraw from the study: You can end your participation by closing the browser window. If you want to withdraw your participation after completing a session, please contact XXX. It is only possible to withdraw up to 2 months after the end of participation. it is not possible to withdraw after the publication of the data.

Questions? For questions, concerns, or complaints, please contact XXX.

A brief introduction to Personal Values

Schwartz defines values as “deeply rooted, abstract motivations that guide, justify or explain attitudes, norms, opinions and actions” (Schwartz, 2003). Furthermore, he adds that values are beliefs linked to personal feelings and desirable goals. Values are ordered by importance relative to one another, and such relative importance subconsciously guides actions and judgment (Schwartz, 2012). Schwartz proposes the following ten *basic values*:

Self-Direction, Stimulation, Hedonism, Achievement, Power,
Security, Conformity, Tradition, Benevolence, Universalism

This list is composed of values applicable to all cultures and contexts. However, as Schwartz points out, not all values are relevant in all contexts. Furthermore, such values are described in a rather general fashion, in order to be applicable to all contexts and domains.

Personal Values in this experiment

The Schwartz value list is an example of a list of general values (other such lists of general values also exist). In this project, we aim at crafting a value list specific to a context of discussion.

To better understand the concept of context-specific values, consider the Schwartz value of *security*. Now, think about what security means in the context of “driving a car” vs. “browsing the internet.” In the context of “driving a car” security may entail *safety* and in the context of “browsing the internet” security may entertain *privacy*. We treat safety and privacy as context-specific values.

Further, the meaning of a value can also be context specific. For example, the value of ‘privacy’ may mean different things when “browsing the Internet” vs. “walking through public spaces”. We would like you to capture such context-specific meanings via a list of *keywords* for each value. The keywords are intended to represent *triggers* for the value in the domain: words or short expressions which, when read in the context, lead you to think about that value. For example, you can define the value of privacy in two different contexts as follows.

- *Value*: Privacy
  Context: browsing the Internet
  Keywords: anonymity, data collection, third-party cookies
- *Value*: Privacy
  Context: walking through public spaces
  Keywords: cameras, facial recognition, personal identification

References:

Schwartz, Shalom H. "A proposal for measuring value orientations across nations." *Questionnaire package of the european social survey* 259.290 (2003): 261.
Schwartz, Shalom H. "An overview of the Schwartz theory of basic values." *Online readings in Psychology and Culture* 2.1 (2012): 2307-0919.

An overview of the Participatory Value Evaluation (PVE) method

Participatory Value Evaluation (PVE) is a novel survey method aimed at measuring the preferences for government policies of a group of citizens (Mouter et al., 2019). In an online environment, participants analyze policy options that the government considers, accompanied by qualitative and quantitative consequences that each one entails. Upon choosing the preferred option(s), participants are invited to motivate each of their choices with a bulk of text which we here refer to as *motivation*.

Motivations are typically value-laden sentences written to justify choices. Their analysis offers valuable insight into the values held by survey participants in a specific context. We invite you to read such motivations and create a list of context-related values, guided by an NLP-supported method.

PVE on Lifting corona measures in the Netherlands

A PVE for understanding participants' preferences on lifting COVID-19-related measures was conducted in the Netherlands between 29/04/2020 and 06/05/2020, when partial lockdown measures were in place in the Netherlands to limit the spread of COVID-19. The government had multiple plans for lifting such measures in the following weeks and months, and wanted to gauge the opinion of the citizens on the subject. Each proposed option came with an additional pressure on the healthcare system as a consequence. The eight proposed relaxation measures are listed below. The numbers in the parenthesis indicate the additional pressure on the healthcare system from a choice.

- Nursing and care homes allow visitors. (10-25%)
- Businesses open again, except for hotels, restaurants, cafes and contact professions (barbers, beauticians, etc.). (6-15%)
- Employees in contact professions (barbers, beauticians, etc.) go back to work. (8-15%)
- Young people may come together in groups. (4-8%)
- All restrictions are lifted for people who are immune. (10-20%)
- Restrictions are lifted in Friesland, Groningen and Drenthe (Northern regions less affected by the virus). (15-30%)
- Social contact within families is allowed again. (6-15%)
- Hotels, restaurants, cafes and entertainment industry re-open. (15-25%)

The participants could choose multiple options, with a limit of maximum 50% additional pressure on the healthcare system. Subsequently, they were asked to motivate each of the chosen options.

The question for you is:
What personal values are relevant in the context of relaxing COVID-19 measures?

PVE on South-West Friesland energy transition

The South-West Friesland (the Netherlands) energy transition PVE was organized by the municipality of South-West Friesland in the scope of transition to renewable energy, with the goal of reaching 50% of renewable energy generation by 2030. The municipality did not have a concrete plan yet, but proposed the following six options to its citizens:

- The municipality takes the lead and unburdens you.
- Inhabitants do it themselves.
- The market determines what is coming.
- Large-scale energy generation will occur in a small number of places (as opposed to being distributed).
- Betting on storage (South-West Friesland becomes the battery of the Netherlands).
- Become an energy supplier in the Netherlands (South-West Friesland helps the rest of the country in generating more sustainable energy).

Citizens were invited to distribute 100 points among the mentioned options, assigning proportionally more points to the preferred options. Subsequently, they were asked to motivate each of the chosen options.

The question for you is:
What personal values are relevant in the context of a municipality's attempts to transition to renewable energy?

References:

Mouter, Niek and Koster, Paul and Dekker, Thijs, An Introduction to Participatory Value Evaluation (December 15, 2019). Tinbergen Institute Discussion Paper 2019-024/V, Available at SSRN: https://ssrn.com/abstract=3358814 or http://dx.doi.org/10.2139/ssrn.3358814

Exploration

The exploration phase is to be performed by you, expert, individually. You will start with an empty list of values. You will be shown the choices participants made and, importantly, the motivations they provided for making those choices, sequentially. We ask you to annotate values as described below.

Exploration workflow

Please follow the flowchart depicted in Figure 1. Here additional details are provided for the components outlined in the flowchart:

- **Is the value list complete?** You start with an empty list, which you populate over time. Please refer to the section “When to stop?” to verify whether your list is complete.
- **Read new motivation:** Click on “*Next (Farther) Motivation*” to read a new participant’s motivation; you see the original Dutch text, the translated English version and the choice for which it was written. You can click on a “*Next Motivation*” button only after having annotated the motivation you are currently shown. You can either annotate with an action (value / keyword addition / deletion), or by indicating why no action is taken on the motivation, by clicking on one of the three red buttons (as explained below).
- **Value behind motivation?** Is there a value related to the motivation? While trying to identify a value, please answer these questions:
    - Can you complete the following sentence: “The participant chose <option> in this PVE because <value> is important to her.”?
    - Can the value be compared to other values in order of importance?

  If you answered ‘yes’ to both questions, please add the identified value to the values list (if not already present).
- **Why no value behind motivation?** it is possible that no value is related to the motivation, or that the motivation is incomprehensible (either due to a bad translation or to unclear original text). In such cases, click on the corresponding red button before clicking on “*Next Motivation*”.
- **Keywords for value in motivation?** By reading the motivation, can you think of keywords for the value behind the motivation? Keywords may or may not appear in the motivation, but should be directly inspired by the motivation.
- **Add value / keywords to the list**: add the desired value and/or keywords by simply entering them in the indicated slots and then clicking the “*Add*” button on the side. Both value and keywords may be composed of multiple words or expressions, such as ‘third-party cookies’.
- **Read suggested additional keywords**: upon adding a value and/or keywords, the algorithm will show additional words similar to the value and/or keywords: you may then decide to add one of such words to the value keywords.
- **Value / keywords already in my list**: a new motivation may not contain any new information with respect to your list of values and keywords. In such a case, click on the “*Value already annotated*” red button before clicking on “*Next Motivation*”.

Please write value and keywords in the *value list* below the motivations, which will always be visible and editable. At any moment, you can add or remove a value or a keyword.

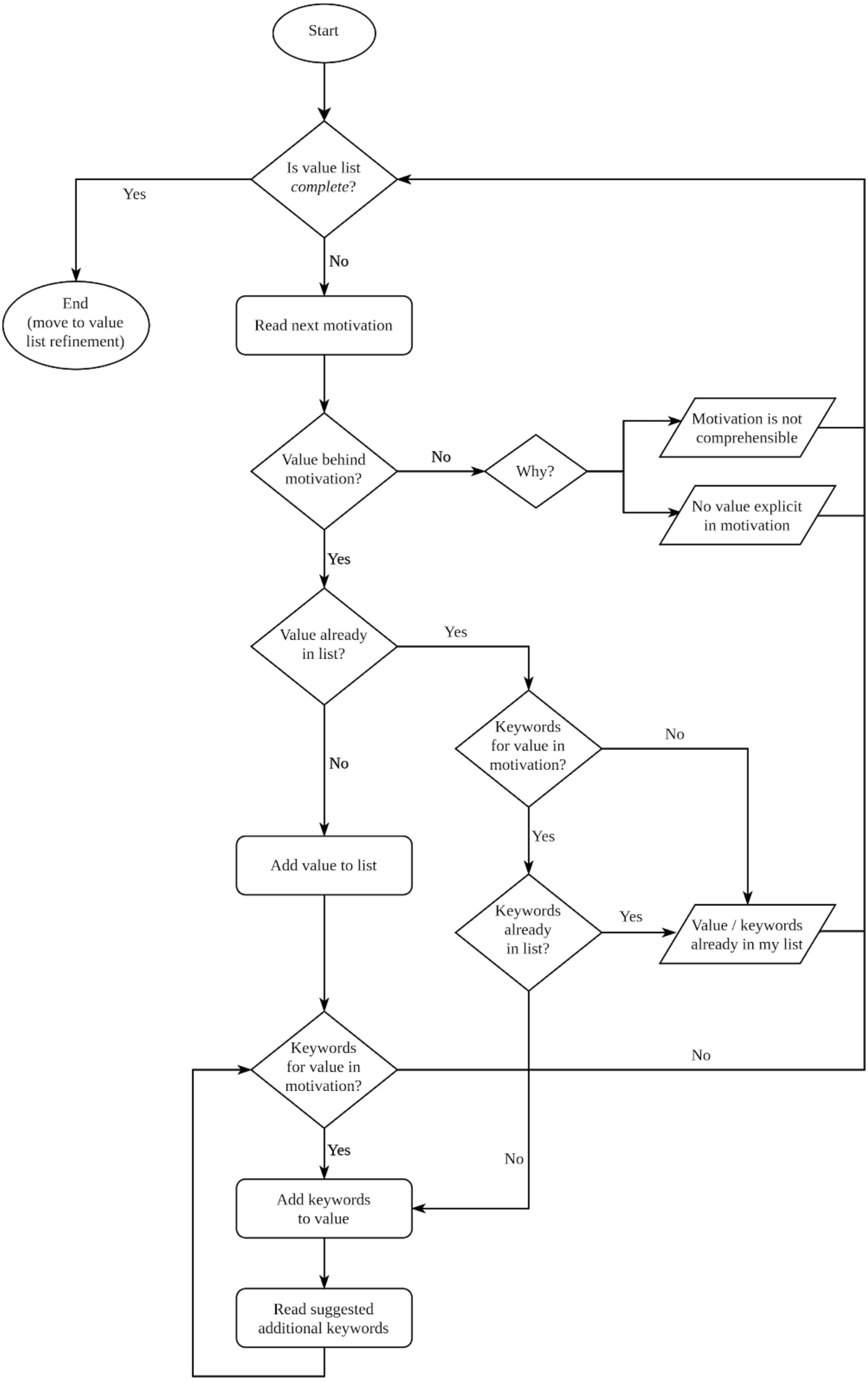

Figure 1: Annotation pipeline during the exploration phase.

Here are two guiding examples of value annotation:

- Next motivation:
    - Read motivation: “I don’t want to be spied by cameras while I walk in the streets of my city. I want to be by myself.” for choice: “All public cameras should be easily identifiable.”
    - Add value ‘privacy’?
    - Insert in sentence: “The participant chose the project ‘All public cameras should be visibly identifiable’ because ‘privacy’ is important to her.”
    - Privacy can be compared to other values (such as safety) by importance.
    - Add value ‘privacy’.
    - Add keywords: ‘spying’, ‘cameras’, ‘by myself’.
    - After adding such value and keywords, the following keywords are suggested: ‘confidentiality’, ‘intimacy’, ‘secrecy’, ‘espionage’, ‘eavesdropping’
    - Add ‘eavesdropping’ to the list of keywords.
- Next motivation:
    - Read motivation: “That’s how it should already be!” for choice: “All public cameras should be visibly identifiable.”
    - Value: none (there are values related to the project choice, but not explicitly in the motivation)

Progress plot

You can track your progress via the *progress plot*. The plot contains a bar per each read motivation. The bar height indicates how the motivation differs from the previously read motivations and the annotated values and keywords; the taller the bar, the more different the motivation. The bars are colored according to the action(s) performed upon reading the motivation, as detailed in the legend.

When to stop?

Please continue until you deem that, based on the inputs you have read so far, no new values will emerge (i.e., the value list is *complete*). You can track the progress of your actions via the progress plot. When you see that your value list has not changed over the last several rounds, you can decide to stop.

Value list refinement

After having decided that the list is complete, please make sure that the values you added are sufficiently distinct one from another and clearly defined by their keywords (i.e., the value list is *well-defined*). By clicking on the *“Next (Similar) Motivation”* button besides each of the values you added, you will be able to see a motivation which, according to the algorithm, is similar to the value. By reading this motivation, you can get inspiration to add additional keywords to the value in order to further characterize it and distinguish from the other values in your list. Upon annotation of the motivation (as described in the previous section), you can click again on the button to see new similar motivations.

Consolidation

Welcome to the consolidation phase! Previously, in the exploration phase, you and other experts, each created a list of values with keywords. Now, you are invited to discuss and *consolidate* the results of your explorations. It is likely that the lists you created in exploration overlap, i.e., they may have similar values and keywords. Such values and keywords are to be consolidated into one list of values with keywords in the consolidation phase.

At first, a draft list is created as the union of the individual value lists. Then, you can fetch a pair of most similar values (as determined by an algorithm) from the draft list and consolidate the two values in this pair. The actions you can take to consolidate a pair of values are explained in the next section. Then, you can fetch and consolidate the next pair of most similar values. You can repeat this process until you deem that the draft list does not require anymore consolidation.

Consolidation workflow

Please follow the flowchart depicted in Figure 1. Additional details for the components outlined in the flowchart are provided below.

- **Does the list need more consolidation?** At the beginning of the phase, let yourself be guided by the algorithm suggestions; as soon as no actions (merge or additions) are taken on several consecutive suggestions, inspect the draft value list and determine if it requires further consolidation. Please refer to the following section for a detailed explanation of the termination criterion. The following steps apply if you wish to consolidate further.
- **Fetch a new value pair.** By clicking on the "*Next pair*" button, the algorithm will provide the pair of most similar values from the draft list. Alternatively, you can manually select the next pair by clicking on the "*Pick next pair*" button and selecting the two values to be shown. The following steps guide you in consolidating the two values in the shown pair.
- **Authors explain values**. The experts who originally annotated the values are invited to give a brief overview of their reasoning to the other experts.
- **Want to read trigger motivations?** In order to refresh your memory, you can read the motivation(s) which led to the annotation of given values and keywords, by clicking on the "*Show*" button present on the top-right corner of the shown values.
- **Consolidate values**. The experts in the group should discuss whether the two values overlap. For example, discuss whether the two values have a similar name, whether the keywords overlap, whether the explanation of the values are similar, and so on. Then, you can perform one of the following actions.
    - **Merge the two values**: If you deem the two values as similar, merge them into one value. Upon merging, a new value is created with the name to be chosen by you (the new value name may or may not be the name of one of the two merged values); the keywords of this new value are the union of the keywords of the two merged values. You can update the keywords. The two originally suggested values are then deleted.
    - **Update one or both values**: If you deem the two values as distinct, you may update the name and/or keywords of one or both values to make the distinction clearer.

- **Take no action**: If you deem the two values as sufficiently distinct as is, you may take no action. The value pairs that were shown but not edited will not be shown again.

Important: The full value list will always be accessible and editable. At any moment you can merge values, add or delete a value, add or delete a keyword.

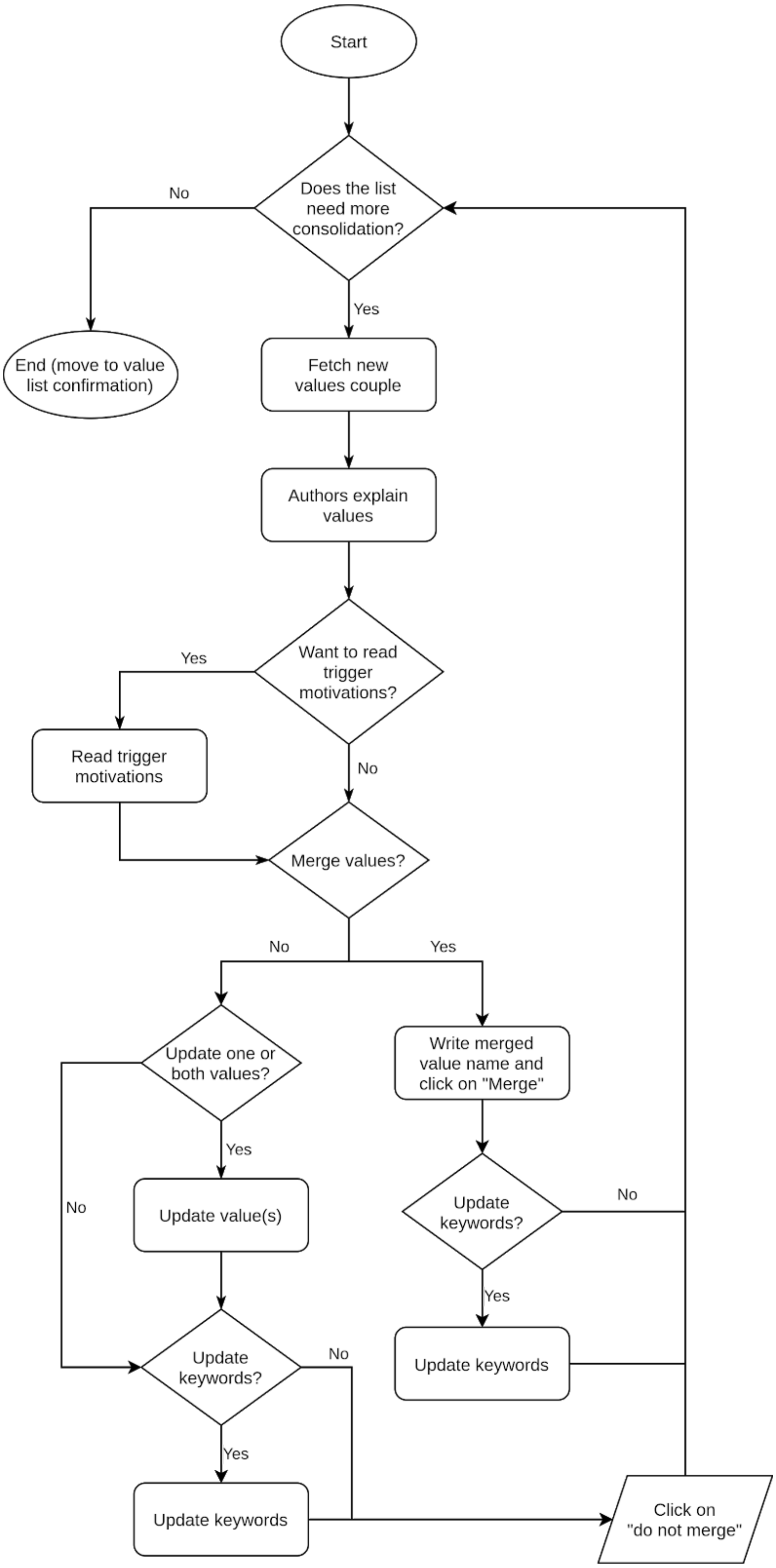

Figure 1: Consolidation workflow.

Here is a guiding example:

- The two following values are proposed:
  - Value: privacy
    Keywords: surveillance, cameras, facial recognition
  - Value: anonymity
    Keywords: cameras, identification
- The values authors explain their reasoning.
- The values are merged into one:
  - Value: privacy
    Keywords: surveillance, cameras, facial recognition, identification

Here is another guiding example:

- The two following values are proposed:
  - Value: privacy
    Keywords: surveillance, cameras, facial recognition
  - Value: safety
    Keywords: cameras, theft, crime
- The values authors explain their reasoning.
- The values are not merged.

Progress plot

You can track your progress via the *progress plot*. The plot contains a bar per each seen value pair. The bar height indicates how different the two values are; the taller the bar, the more different the values. The bars are colored according to the action(s) performed upon reading the value couple, as detailed in the legend.

When to stop?

Stop when the values in the draft list are sufficiently distinct one from another and clearly defined by their keywords (i.e., they are *well-defined*). You can track your progress via the progress plot, and at any moment look at the full list of values and keywords.

Value list reflection

The final step in the consolidation phase is to confirm the values in the consolidated list. To do so, **ask critical questions** on each value in the value list. Schwartz describes the features of all values indicated below. The experts in the group should ask whether each value in the list has each of these features or not.

- Values are beliefs linked inextricably to affect. When values are activated, they become infused with feeling.
- Values refer to desirable goals that motivate action.
- Values serve as standards or criteria. Values guide the selection or evaluation of actions, policies, people, and events.

- Values are ordered by importance relative to one another. People's values form an ordered system of priorities that characterize them as individuals.
- The relative importance of multiple values guides action. Any attitude or behavior typically has implications for more than one value.

Important: Schwartz also includes an additional feature that "values transcend specific actions and situations." However, since we are interested in values for a specific context, this feature may not apply. Nonetheless, the values in your list can transcend different actions within the same context.

As you confirm each value, you should also create a shared *definition of the value*, agreed by all experts in the consolidation group. In order to do so, we draw inspiration from Schwartz and bind each value to a *defining goal* (Schwartz, 2012). A defining goal characterises what "holding a value" means. That is, if a person holds a value, they are likely to have the corresponding goal in life.

The following are the defining goals for the 10 Schwartz values.

1. *Self-Direction*: Independent thought and action--choosing, creating, exploring.
2. *Stimulation*: Excitement, novelty, and challenge in life.
3. *Hedonism*: Pleasure or sensuous gratification for oneself.
4. *Achievement*: Personal success through demonstrating competence according to social standards.
5. *Power*: Social status and prestige, control or dominance over people and resources.
6. *Security*: Safety, harmony, and stability of society, of relationships, and of self.
7. *Conformity*: Restraint of actions, inclinations, and impulses likely to upset or harm others and violate social expectations or norms.
8. *Tradition*: Respect, commitment, and acceptance of the customs and ideas that one's culture or religion provides.
9. *Benevolence*: Preserving and enhancing the welfare of those with whom one is in frequent personal contact (the 'in-group').
10. *Universalism*: Understanding, appreciation, tolerance, and protection for the welfare of all people and for nature.

Upon termination of the consolidation procedure according to the described criterion, please add the defining goal(s) to each value present in the final list by using the "*Add defining goal*" button.

Note that the defining goals don't have to be generic (as in the defining goals of Schwartz values). In contrast, the defining goals can be specific to the context in which the values apply. For instance, in the context of an internet application, the defining goal for the value of privacy can be as follows.

- *Privacy*: Being able to control the extent to which one's personal information is shared with others.

References:

Schwartz, Shalom H. "An overview of the Schwartz theory of basic values." *Online readings in Psychology and Culture* 2.1 (2012): 2307-0919.
