# Supplementary material for: What values should an agent align with? An empirical comparison of general and context-specific values
Source: Auton Agent Multi Agent Syst. 2022 Mar 28;36(1):23. doi: 10.1007/s10458-022-09550-0 (PMC8958938; doi:10.1007/s10458-022-09550-0)
Supplement: Supplementary file 3 — Electronic supplementary material 3 (PDF 94 kb) [file 10458_2022_9550_MOESM3_ESM.pdf]

Informed Consent Form

Purpose of this research study: In this study, we aim at creating a list of context-related personal values, based on input provided by participants to a survey.

What you will do in the study: You will annotate personal values based on written input provided by participants to a survey. Additional details will follow.

Time required: 45 minutes.

Risks: There are no risks anticipated in this study. However, in case of doubts or concerns, do not hesitate to contact the researchers.

Benefits: You will not directly benefit from participating in this study. After you completed the survey you will be rewarded as stated on Prolific.

Privacy and confidentiality: Should you agree to take part, your participation will be completely confidential. You will be identified only through your Prolific ID. All information gathered in the survey will be stored securely in compliance with the standards set by the European Union General Data Protection Regulation (GDPR). No one outside the research group will have access to the data. Demographic data will be kept by the research group until the analyses are finalized, at the latest in September 2021. By this date, demographic data will be aggregated and individual demographic information will be destroyed. Upon analysis and publication, the aggregated demographic data and the annotation data will be stored and made available open access for other researchers to analyse. This study is approved by the YYY Ethics Committee.

Right to withdraw from the study: Participation in the study is completely voluntary. If at any time you do not wish to continue your participation, you are welcome to withdraw from the survey without penalty.

How to withdraw from the study: You can end your participation by closing the browser window. If you want to withdraw your participation after completing a session, please contact XXX. It is only possible to withdraw up to one week after the end of participation. it is not possible to withdraw after the publication of the data.

Questions? For questions, concerns, or complaints, please contact XXX.

Instructions

Thank you for joining the Axies evaluation! Please find the instructions below.

Introduction

We have a list of personal values relevant to a specific context of discussion, based on the input that participants provided to a survey (additional details follow). Here, we are asking you to evaluate clarity and completeness of the value list.

Value list

Values are abstract motivations that justify opinions and actions, linked to personal feelings and desirable goals. In this experiment, values are represented by a name, a defining goal and a set of keywords. Defining goals and keywords are used to describe the value and capture its meaning in the context under discussion. For example, in the context of browsing the internet, the value of 'privacy' can be represented as follows:

- Value: privacy
- Keywords: anonymity, data collection, third-party cookies
- Defining goal: Being able to control the extent to which one’s personal information is shared with others.

Survey context (Covid)

In April, Dutch citizens answered a survey about ways for lifting COVID-19-related measures. In this survey, participants were proposed eight different options, and could choose one or more of them (for example, “All restrictions are lifted for immune people” and “Nursing homes allow visitors again”). They were then invited to explain each chosen option with a motivation.

Survey context (Energy)

The South-West Friesland municipality (in the Netherlands) organized a survey in the scope of transition to renewable energy, with the goal of reaching 50% of renewable energy generation by 2030. They offered multiple options to the participants, who could choose one or more of them (such as “The municipality takes the lead” and “The market determines what’s coming”). Participants were then invited to explain each chosen option with a motivation.

Clarity evaluation

Here you can see all values present in the value list, each accompanied by defining goal and keywords. Please indicate how clearly you understand the concept described by each value.

Ambiguity evaluation

You are shown some pairs of values, each accompanied by its defining goal and keywords. Please indicate how *motivationally distinct* (i.e. to which extent can they be distinguished through their defining goals and keywords) you think the two described concepts are, in a 5-points scale. Please keep in mind that values can have overlap, but still be distinct. For instance, the values of ‘achievement’ (personal success through demonstrating competence according to social standards) and ‘power’ (social status and prestige, control or dominance over people and resources) are somewhat similar. However, their distinction comes from the fact that 'achievement' emphasizes the active demonstration of successful performance, whereas 'power' emphasizes the attainment or preservation of a dominant position within the social system.

Opinion annotation

You are sequentially shown the motivations (with the choices) that participants gave in the survey. Please, indicate which value(s) lie behind each motivation (the related survey choice is there just to help you understand the context of the motivation. The value(s) you indicate should be specifically present in the motivation.
On the right side of the screen, you can see the full list of values. For each motivation, you have the following options:

- There is a value lying behind the motivation. You can do one or both the following actions and then click on "Submit":
    - Select from the list the value(s) lying behind the motivation.
    - The value lying behind the motivation is not in the list. Add it in the slot at the bottom of the list. Optionally, you can explain the reason why you added it, and some words from the motivation that are related to it.
- There is no value lying behind the motivation, or the motivation is unclear. Click on "No values" to get the next motivation.
