# Supplementary material for: What values should an agent align with? An empirical comparison of general and context-specific values
Source: Auton Agent Multi Agent Syst. 2022 Mar 28;36(1):23. doi: 10.1007/s10458-022-09550-0 (PMC8958938; doi:10.1007/s10458-022-09550-0)
Supplement: Supplementary file 4 — Electronic supplementary material 4 (PDF 77 kb) [file 10458_2022_9550_MOESM4_ESM.pdf]

Informed Consent Form

Purpose of this research study: In this study, we aim at creating a list of context-related personal values, based on input provided by participants to a survey.

What you will do in the study: You will annotate personal values based on written input provided by participants to a survey. Additional details will follow.

Time required: max 4 hours.

Risks: There are no risks anticipated in this study. However, in case of doubts or concerns, do not hesitate to contact the researchers.

Benefits: You will not directly benefit from participating in this study. After you completed the survey you will be rewarded.

Privacy and confidentiality: Should you agree to take part, your participation will be completely confidential. All information gathered in the survey will be stored securely in compliance with the standards set by the European Union General Data Protection Regulation (GDPR). No one outside the research group will have access to the data. Demographic data will be kept by the research group until the analyses are finalized, at the latest in September 2021. By this date, demographic data will be aggregated and individual demographic information will be destroyed. Upon analysis and publication, the aggregated demographic data and the annotation data will be stored and made available open access for other researchers to analyse. This study is approved by the YYY Ethics Committee.

Right to withdraw from the study: Participation in the study is completely voluntary. If at any time you do not wish to continue your participation, you are welcome to withdraw from the survey without penalty.

How to withdraw from the study: You can end your participation by closing the browser window. If you want to withdraw your participation after completing a session, please contact XXX. It is only possible to withdraw up to one week after the end of participation. it is not possible to withdraw after the publication of the data.

Questions? For questions, concerns, or complaints, please contact XXX.

Instructions

Thank you for joining the Axies Value list evaluation. Please find the instructions below.

Introduction

You will be asked to provide some insights into values with relation to two different contexts. Please read the description below as a small background on the different contexts. The experiment assumes you have some prior knowledge on personal values.

Survey Context (Covid)

A PVE for understanding participants' preferences on lifting COVID-19-related measures was conducted in the Netherlands between 29/04/2020 and 06/05/2020, when partial lockdown measures were in place in the Netherlands to limit the spread of COVID-19. The government had multiple plans for lifting such measures in the following weeks and months, and wanted to gauge the opinion of the citizens on the subject. Each proposed option came with an additional pressure on the healthcare system as a consequence. The numbers in the parenthesis indicate the additional pressure on the healthcare system from a choice. The participants could choose multiple options, with a limit of maximum 50% additional pressure on the healthcare system. Subsequently, they were asked to motivate each of the chosen options.

Survey Context (Energy)

The South-West Friesland (the Netherlands) energy transition PVE was organized by the municipality of South-West Friesland in the scope of transition to renewable energy, with the goal of reaching 50% of renewable energy generation by 2030. The municipality did not have a concrete plan yet, but proposed six options to its citizens. Citizens were invited to distribute 100 points among the mentioned options, assigning proportionally more points to the preferred options. Subsequently, they were asked to motivate each of the chosen options.

Task description

Your task is to individually annotate, for a set of 47 values, how well each of them would help you in the decision making process in a specific context. In this scenario, you need to imagine yourself in the position of the policy maker. For each value, look up its complete definition as given in the value overview. Please read the full definition, and try to take in the concept of the value.

Next, please give your agreement with the following statement for both contexts each: If I am a policy maker in a specific context, knowing citizens' preferences about this value would help me in making a policy decision in that context. The agreement is on a scale of 1 (strongly disagree) to 5 (strongly agree).

Additionally, please note which (if any) of the options were the most relevant in your thought process. If no specific options come to mind, you can simply leave this empty.

For example, in the context of COVID exit (context), knowing citizens' preferences about mental health (value) would help me in deciding whether to allow visitors to nursing and care homes or not (a policy decision). This I would annotate with a score of a 5: strongly agree
Once the annotation has completed, you will move on to a post-annotation phase (see "Post-annotation").

Post annotation

After annotating your ratings, you will be asked to go back to your ratings and talk about the values where you disagreed most with the other annotator in terms of rating. Discuss why your ratings differ, possibly referring to the options that you found most relevant. After this discussion, you are free to adjust your rating. If you decide to adjust your rating, please note down in 1-2 sentences what caused you to change your rating.
